# CPUY192018, a potent inhibitor of the Keap1-Nrf2 protein-protein interaction, alleviates renal inflammation in mice by restricting oxidative stress and NF-κB activation

**DOI:** 10.1016/j.redox.2019.101266

**Published:** 2019-07-02

**Authors:** Meng-Chen Lu, Jing Zhao, Yu-Ting Liu, Tian Liu, Meng-Min Tao, Qi-Dong You, Zheng-Yu Jiang

**Affiliations:** a State Key Laboratory of Natural Medicines and Jiangsu Key Laboratory of Drug Design and Optimization, China Pharmaceutical University, Nanjing, 210009, China; b Department of Medicinal Chemistry, School of Pharmacy, China Pharmaceutical University, Nanjing, 210009, China

**Keywords:** Keap1-Nrf2 pathway, Protein-protein interaction inhibitors, ROS, NF-κB, Renal inflammation

## Abstract

The Keap1-Nrf2-ARE pathway regulates the constitutive and inducible transcription of various genes that encode detoxification enzymes, antioxidant proteins and anti-inflammatory proteins and has pivotal roles in the defence against cellular oxidative stress. In this study, we investigated the therapeutic potential of **CPUY192018**, a potent small-molecule inhibitor of the Keap1-Nrf2 protein-protein interaction (PPI), in renal inflammation. In human proximal tubular epithelial HK-2 cells, **CPUY192018** treatment significantly increased Nrf2 protein level and Nrf2 nuclear translocation, which enhanced Nrf2-ARE transcription capacity and the downstream protein content in a Nrf2 dependent manner. In lipopolysaccharide (LPS)-challenged human HK-2 cells, **CPUY192018** exhibited cytoprotective effects by enhancing the Nrf2-ARE regulated antioxidant system and diminished the LPS-induced inflammatory response by hindering the ROS-mediated activation of the NF-κB pathway. In the LPS-induced mouse model of chronic renal inflammation, by activating Nrf2, **CPUY192018** treatment balanced renal oxidative stress and suppressed inflammatory responses. Hence, administration of **CPUY192018** reduced kidney damage and ameliorated pathological alterations of the glomerulus. Taken together, our study suggested that small-molecule Keap1-Nrf2 PPI inhibitors can activate the Nrf2-based cytoprotective system and protect the kidney from inflammatory injury, raising a potential application of Keap1-Nrf2 PPI inhibitors in the treatment of inflammatory kidney disorders.

## Highlights

• **CPUY192018** activated Nrf2-ARE pathway to protect against LPS-induced renal inflammation both in cells and *in vivo*.
• **CPUY192018** also inhibited NF-κB involved inflammatory response both in cells and kidney.
• The development of Keap1-Nrf2 PPI inhibitors may create treatment options for kidney diseases with reduced off-target effects.

## Graphical abstract

**Figure:**
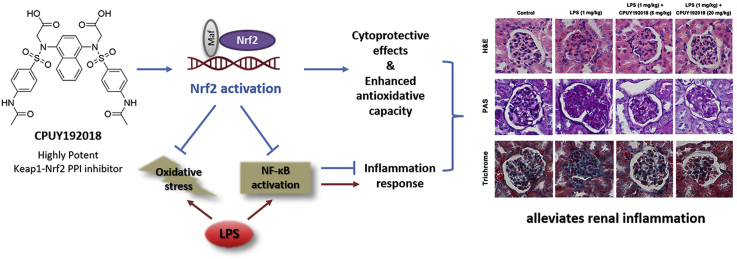

## 1 Introduction

The transcription factor nuclear factor erythroid-2 related factor 2 (Nrf2) is the dominant manager of the cellular defence system [1,2]. By binding to the enhancer sequence in the gene promoter regulatory region that is termed the antioxidant response element (ARE), Nrf2 regulates the expression of a wide array of genes encoding antioxidant proteins, detoxifying enzymes, metabolic alteration enzymes and stress response proteins, most of which play important roles in the cellular defence system, especially in oxidative stress modulation [[3], [4], [5]]. Through regulating this transcriptional network, Nrf2 is able to coordinate a fine-tuned response to various stress conditions and harmful assaults, enabling the cellular microenvironmental homeostasis [6]. Thus, enhancing Nrf2 activity can provide cytoprotection against various chronic and inflammatory conditions [5,7].

There is considerable experimental evidence suggesting that Nrf2 activation can prevent kidney disease progression by preventing oxidative stress, enhancing the metabolic capacity of toxic assaults and suppressing inflammatory conditions [[8], [9], [10], [11], [12]]. Basic research has demonstrated that oxidative stress is a major pathogenic and aggravating factor for chronic kidney disease (CKD) and related complications [13,14]. Enhanced oxidative stress mainly results from overproduction of reactive oxygen species (ROS) in the context of concomitant, insufficient antioxidant pathways [15]. While low-levels of ROS function as signalling molecules for cellular proliferation and vascular homeostasis, an imbalance of ROS generation and elimination in the kidneys leads to necrosis, apoptosis, inflammation, fibrosis, and other disorders that participate in disease processes [16]. Therefore, eliminating oxidative stress and maintaining microenvironmental homeostasis can antagonize pathogenesis and the progression of kidney diseases.

Kelch-like ECH-associated protein 1 (Keap1) is the predominant repressor protein of Nrf2 [17,18]. By acting as an E3 ligase adaptor component, Keap1 facilitates the polyubiquitination of the Nrf2 protein, leading to proteasome-dependent Nrf2 degradation [19,20]. Inhibiting Keap1 activity can hinder the degradation of Nrf2 by ubiquitin-proteasome system, resulting in the accumulation of newly synthesized Nrf2 and its translocation to the nucleus, where it induces the transcription of a battery of antioxidative and cytoprotective genes, ultimately leading to activation of the cell defence system [21,22].

The development of small-molecule Nrf2 activators targeting Keap1 has a long history [23,24]. Most of traditional Nrf2 activators are electrophilic agents which target the reactive cysteine residues in Keap1, and these cysteine residues serve as sensors of the intracellular redox state [25,26]. Oxidative or covalent modification of these cysteine residues cause conformational changes of Keap1, hindering the Nrf2 ubiquitination process [21,27]. Some electrophilic Nrf2 activators undergoing clinical trials have been proven to be beneficial for alleviating kidney diseases. A well-known electrophilic Nrf2 activator, bardoxolone methyl, showed promising outcome in the early phase clinical studies [28]. Unfortunately, a phase III study of bardoxolone methyl was terminated for safety risk. Inhibiting Keap1 by covalent modification of cysteine residues is a validated way to activate Nrf2, but safety and selectivity concerns remain in this method, for that cysteine is ubiquitous in cells.

Recently, discovery of Keap1-Nrf2 PPI inhibitors has emerged as a novel way to activate Nrf2 [9]. Unlike traditional Nrf2 activators, the Keap1-Nrf2 inhibitors can competitively and directly disrupt the Keap1-Nrf2 PPI by non-covalent interactions [29,30]. The advantage of a direct inhibitor of the Keap1-Nrf2 interaction is a more selective mechanism of action with lower propensity for off-target properties through activation or repression of other pathways [31], and such an approach may be beneficial for further applications. With the development of the structural and mechanistic knowledge of the Keap1-Nrf2 system, various Keap1-Nrf2 PPI inhibitors have been developed as potential therapeutics against chronic and inflammatory conditions, including pulmonary inflammation [32], cardiomyopathy [33] and myocarditis [34]. However, the cytoprotective effects and therapeutic potential of the Keap1-Nrf2 inhibitors on inflammatory kidney disease remains unclear.

Our group previously discovered and identified the first Keap1-Nrf2 inhibitor effective at nanomolar concentrations, **CPUY192002** [35]. Subsequent solubility optimization of **CPUY192002** by medicinal chemistry methods resulted in **CPUY192018** with better physicochemical properties. **CPUY192018** showed potent Nrf2 activation effects both *in vitro* and *in vivo*, and it has been proven to be useful in the dextran sodium sulphate-induced experimental colitis model [36]. In this study, we evaluated the cytoprotective effects of **CPUY192018** against lipopolysaccharide (LPS)-induced injury in HK-2 human proximal tubular cells and investigated the therapeutic potential of a Keap1-Nrf2 PPI inhibitor in an experimental renal model induced by LPS. Our results proved that **CPUY192018** can activate Nrf2-dependent antioxidative pathways and inhibit NF-κB involved inflammatory response, which antagonized the LPS-induced chronic renal inflammation both in the HK-2 cells and *in vivo*. Therefore, our study has identified that the Keap1-Nrf2 inhibitors may be potential treatment options for chronic kidney diseases with reduced off-target effects.

## 2 Materials and methods

### 2.1 Cell culture conditions

HepG2 cells stably transfected with a luciferase reporter (HepG2-ARE-C8) were kindly provided by Professor Dr. A. N. Tony Kong (Rutgers University, Piscataway, NJ) and Professor Rong Hu (China Pharmaceutical University, Nanjing). Cells were maintained in modified RPMI-1640 medium (GiBco, Invitrogen Corp., USA) with 10% fetal bovine serum (FBS) (GiBco, Invitrogen Corp., USA) and penicillin/streptomycin in a 37 °C incubator with 5% CO_2_. Renal proximal tubular epithelial cell line, HK-2 (INCELL, San Antonio, TX) were cultured in Dulbecco's Modified Eagle Medium (Life TechnologyTM, 1645798) supplemented with 10% (v/v) FBS and penicillin/streptomycin. The cells were maintained at condition of 37 °C and 5% CO_2_.

### 2.2 ARE-luciferase activity assay

The experimental procedures were carried out as reported previously [37]. Generally, HepG2-ARE-C8 cells were plated in 96-well plates at a density of 4 × 10^4 cells/well and incubated overnight. The cells were exposed with different concentrations of test compounds, with t-BHQ serving as positive control, DMSO as a negative control, and the luciferase cell culture lysis reagent as a blank. After 12 h of treatment, the medium was removed and 100 μL of cold PBS was added into each well. Then the cells were harvested in the luciferase cell culture lysis reagent. After centrifugation, 20 μL of the supernatant was used for determining the luciferase activity according to the protocol provided by the manufacturer (Promega, Madison, WI). The luciferase activity was measured by a Luminoskan Ascent (Thermo Scientific, USA). The data were obtained in triplicates and expressed as fold induction over control.

### 2.3 Western blotting

*Anti*-Nrf2 (ab62352), *anti*-Ubiquitin (ab134952), *anti*-IL-1β (ab45692), *anti*–NF–κB p65 (ab16502), anti-SOD1 (ab13498), *anti*-GPx1 (ab108427) and anti-catalase (ab76110) antibodies were purchased from Abcam Technology (Abcam Technology, England). *Anti*-phospho–NF–κB p65 antibodies (CST #3031), *anti*-phospho-IκBα (CST #2859) and *anti*-phospho-IKKβ (CST #2694) antibodies were purchased from Cell Signalling Technology (Cell Signalling Technology, USA). Anti–HO–1 (SC-136960) and *anti*-NQO1 (SC-271116) antibodies were purchased from Santa Cruz Biotechnology (Santa Cruz, CA, USA). *Anti*-β-actin (60008-1-lg), anti-Histone-H3 (17168-1-AP) and *anti*-GCLM (14241-1-AP) antibodies were purchased from Proteintech Group (Proteintech Group, USA). Isolation of cell fractions and Western blotting were performed as detailed previously [36].

### 2.4 Immunofluorescence

HK-2 cells were treated with CPUY192018 (10 μM) at indicated times, then incubated at 4 °C overnight with Nrf2 primary antibodies (abcam, UK). After washing with PBS, cells were incubated at 37 °C for 1 h with FITC-labeled secondary goat anti-rabbit IgG antibody (Life Technology). Cells were then stained with fluorochrome dye DAPI (Santa Cruz Biotechnology, Santa Cruz, CA) to visualize the nuclei and observed under a laser scanning confocal microscope (Olympus Fluoview FV1000, Japan) with a peak excitation wave length of 570 nm and 340 nm.

### 2.5 Quantitative real time RT PCR

The experimental procedure of quantitative real-time RT-PCR was previously reported [3]. Primers used for qRT-PCR are listed as follows: Nrf2 (Sense primer: AACCACCCTGAAAGCACGC, Antisense primer: TGAAATGCCGGAGTCAGAATC); HO-1 (Sense primer: ATGGCCTCCCTGTACCACATC, Antisense primer: TGTTGCGCTCAATCTCCTCCT); NQO-1 (Sense primer: CGCAGACCTTGTGATATTCCAG, Antisense primer: CGTTTCTTCCATCCTTCCAGG); GCLM (Sense primer: TTGGAGTTGCACAGCTGGATTC, Antisense primer: TGGTTTTACCTGTGCCCACTG).

### 2.6 Transfection of small interfering RNA (siRNA)

Predesigned siRNA against human Nrf2 (catalogue no. 37030) and control scrambled siRNA (catalogue no. 37007) were purchased from GenScript (GenScript, China). HK-2 cells were plated at a density of 6 × 10^5 cells per 60 mm dish. Cells were transfected with 50 nM siRNA against Nrf2 or 50 nM scrambled duplex using Lipofectamine 2000 (Invitrogen). After 24 h incubation, fresh medium was added, and the cells were cultured for another 48 h. The cells were then treated with compounds for an additional 10 h and lysed for use in qRT-PCR.

### 2.7 Detection of SOD, GSH-Px, CAT and MDA activities and the ratio of GSH/GSSG

The activities of SOD (Total Superoxide Dismutase Assay Kit with WST-8, S0101, Beyotime, China), GSH-Px (Total Glutathione Peroxidase Assay Kit, S0058, Beyotime, China), CAT (Catalase Assay Kit, S0051, Beyotime, China) and MDA (MDA Detection Kit, S0131, Beyotime, China) were determined using the corresponding detection kits according to the manufacturer's instructions. The ratio of GSH/GSSG was evaluated using commercially available kit according to the manufacturer's instructions (GSH and GSSG Assay Kit, S0053, Beyotime, China).

### 2.8 Living Cell Microscopy

HK-2 cells were seeded in 6-well plates at the density of 70–80% confluence per well for overnight incubation. Then the cells were treated with test samples for indicated time. After treatment, cells were washed once with 2 mL of 10% PBS and stained with 10 μM cH_2_DCF-DA (S0033, Reactive Oxygen Species Assay Kit, Beyotime, China) in the dark at 37 °C for 20 min in DMEM medium free with FBS. Analysis was done with a fluorescence microscope (OLYMPUS DP72, Japan) equipped with a U-RFL-T power supply.

### 2.9 Cell viability assay

The cell viability was determined using the MTT assay. Briefly, HK-2 cells in logarithmic phase were seeded at the density of 70–80% confluence per well in 96-well plates at 37 °C with 5% CO_2_ for overnight incubation. After treatment, 20 μL of 5 mg/mL MTT was added and the cells were incubated for 4 h at 37 °C. The supernatant was discarded and 150 μL of DMSO was added to each well. The mixture was shaken on a mini shaker at room temperature for 5 min and the spectrophotometric absorbance was measured by Multiskan Spectrum Microplate Reader (Thermo, USA) at 570 nm and 630 nm. Triplicate experiments were performed in a parallel manner for each concentration point and the results were presented as the means ± SEM. The net A570nm-A630nm was taken as the index of cell viability. The net absorbance from the wells of cells cultured with DMSO was taken as the 100% viability value. The percent viability of the treated cells was calculated by the formula: viability (%) = (A570nm-A630nm) treated/(A570nm-A630nm) control × 100%.

### 2.10 Flow cytometric detection of apoptosis

HK-2 cells in logarithmic phase in a 6 well tissue culture plate were treated with test samples for indicated time. Then they were harvested, washed and resuspended with PBS. Apoptotic cells were determined with an FITC Annexin V Apoptosis Detection Kit (Beyotime, China) according to the manufacturer's protocol. Briefly, cells were washed and incubated for 15 min at room temperature in the dark in 100 μL of 1 × binding buffer containing 5 μL of Annexin V-FITC and 5 μL of PI. Apoptosis was analyzed by FAC Scan laser flow cytometer (Guava easycyte HT, Millipore, CA).

### 2.11 Cell cycle analysis

The ratio of HK-2 cells in the G0/G1, S and G2/M phases of cell cycle was determined by their DNA content. After treatment, cells were then harvested, washed twice with cold PBS, and fixed with 75% ice-cold ethanol overnight. Fixed cells were washed twice with cold PBS and incubated with 5 μL of 100 μg/mL RNase A for 30 min at room temperature. After incubation, the cells were stained with 50 μg/mL PI for 30 min in the dark and analyzed by flow cytometry. Untreated cells were used as a control.

### 2.12 IL-1β, IL-18, IL-6, TNF-α and NO production

Levels of IL-1β (IL-1β (h) ELISA kit, EK0392, Boster), IL-18 (IL-18 (h) ELISA kit, EK0864, Boster), IL-6 (IL-6 (h) ELISA kit, EK0410, Boster), TNF-α (TNF-α (h) ELISA kit, EK0525, Boster) and NO production (Nitrate/Nitrite Assay Kit, S0023, Beyotime, China) were evaluated in human HK-2 cell culture supernatant using commercially available kits according to the manufacturer's instructions.

### 2.13 NF-κB localization by immunofluorescence

The detection of NF-κB nuclear translocation was carried out following the instruction of the kit (NF-κB Activation, Nuclear Translocation Assay Kit, SN368, Beyotime, China). Briefly, HK-2 cells were pretreated with **CPUY192018** for 10 h and then exposed to 200 ng/mL LPS for an additional 6 h at 37 °C. After washing with PBS, cells were fixed with 4% paraformaldehyde, permeabilized with 0.1% Triton X-100 for 15 min and blocked with 5% BSA for 2 h at room temperature. The cells were then incubated overnight with the primary antibody against the p65 subunit of NF-κB at 4 °C, washed in PBS, and incubated with Cy3-labeled secondary antibody for 2 h at room temperature. Finally, the cells were stained with 2 μM DAPI for 15 min, and then observed using a fluorescence microscope.

### 2.14 Animal experiments

#### 2.14.1 Animals

Animal studies were conducted according to protocols approved by Institutional Animal Care and Use Committee of China Pharmaceutical University. All animals were appropriately used in a scientifically valid and ethical manner. Female C57BL/6 mice (Comparative Medicine Centre, Yangzhou University, China), 6–8 weeks of age weighing 18–20 g, were acclimatized under a 12 h light/dark cycle at 22 °C and 60% humidity for 3 days before the experiments and fed with a standard laboratory rodent diet and water. Mice were given free access to diet and water during the course of experiments. Food intake and body weight were measured daily.

The animals were randomly assigned to one of the four treatment groups (8 animals in each group): (A) Control group; (B) LPS model group (the mice received 1 mg/kg LPS through i.p. Injection); (C) LPS + **CPUY192018** (5 mg/kg) group (the mice received 1 mg/kg LPS together with 5 mg/kg **CPUY192018** through i.p. Injection); and (D) LPS + **CPUY192018** (20 mg/kg) group (the mice received 1 mg/kg LPS together with 20 mg/kg **CPUY192018** through i.p. Injection). Briefly, LPS was administered intraperitoneally at a dose of 1 mg/kg body weight every day for 8 consecutive weeks. Two days prior to the first dose LPS injection, a daily dose of **CPUY192018** (5 mg/kg for Group C, 20 mg/kg for Group D) or corn oil (Group A) was administered *via* the intraperitoneal route until the sacrifice of the mice at week 8. Renal cortical tissues and blood samples were collected at week 8 and stored appropriately for further analysis.

#### 2.14.2 Histopathologic scoring

Sections of sham-operated, the LPS-induced and **CPUY192018**-treated kidneys were fixed in 10% neutral buffered formalin and then embedded in paraffin. Histopathologic scoring was performed using a blind method and the score was assessed by grading tubular necrosis, loss of brush border, cast formation, and tubular dilatation as follows: 0, none; 1, 10%; 2, 11–25%; 3, 26–45%; 4, 46–75%; and 5, 76% [38].

#### 2.14.3 Renal morphology assessment and immunohistochemical analysis

Tissue sections from paraffin-embedded kidney were stained with haematoxylin-eosin (HE), periodic acid Schiff (PAS), and Masson's trichrome. Images were captured by CCD camera system (Advanced Microscopy Techniques, Woburn, MA). The dissected kidney tissues were prepared for IHC analysis of the expression patterns of Nrf2, HO-1, NQO-1, GCLM, p65, p-p65 and IL-1β. IHC analysis was performed as described previously [36].

#### 2.14.4 IL-1β, IL-18, IL-6, TNF-α and NO production in blood serum

Serum levels of IL-1β (IL-1β (m) ELISA kit, EK0394, Boster), IL-18 (IL-18 (m) ELISA kit, #EMC011, NeoBioscience), IL-6 (IL-6 (m) ELISA kit, EK0411, Boster), TNF-α (TNF-α (m) ELISA kit, EK0527, Boster) and NO production (Nitrate/Nitrite Assay Kit, S0023, Beyotime, China) were evaluated using commercially available kits according to the manufacturer's instructions.

## 3 Results

### 3.1 CPUY192018 elevated the protein level of Nrf2 and promoted Nrf2 nuclear translocation in HK-2 cells

**Fig. 1:**
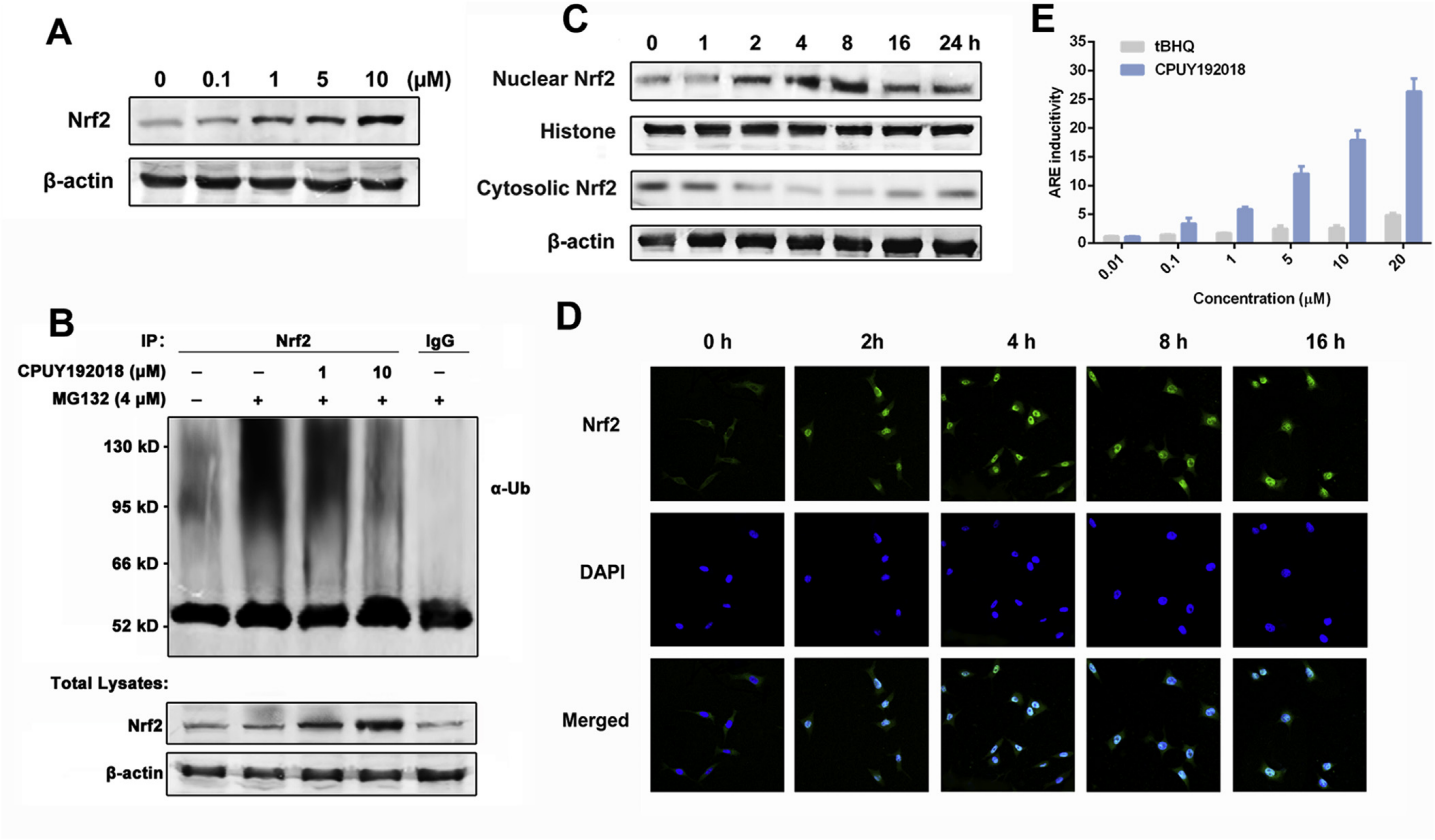
**CPUY192018 elevated the protein level of Nrf2 and promoted Nrf2 nuclear translocation in HK-2 cells**. (A) Effects of **CPUY192018** on the induction of the Nrf2 protein expression. (B) Effects of **CPUY192018** on the ubiquitination of Nrf2 in HK-2 cells. (C) Effects of **CPUY192018** on the nuclear translocation of the Nrf2 protein. At various time points after the treatment with **CPUY192018** (10 μM), nuclear and cytoplasmic cell extracts were prepared from HK-2 cells and subjected to Western blot analysis. Histone and β-actin served as markers for nuclear and cytosolic Nrf2 proteins, respectively. (D) Immunofluorescence staining of Nrf2 at the indicated times in HK-2 cells treated with 10 μM **CPUY192018**. Nrf2 and the nuclei were labeled with FITC and DAPI, respectively. The bars indicate the magnification (10 μm). (E) ARE induction by **CPUY192018** and t-BHQ in the HepG2-ARE-C8 cells. Cells were exposed to the compounds or DMSO for 12 h. The activities are shown as the ratio to the DMSO control. The values shown are the means ± SEM (n = 3 independent observations).

Previously, we have reported the identification of nanomolar Keap1-Nrf2 PPI inhibitor **CPUY192018**, which can mimic the binding pattern of Nrf2 and has a strong binding affinity for Keap1, with an IC_50_ of 14.4 nM in a fluorescence polarization assay and a *K*_*d*_ value of 39.8 nM in an isothermal titration calorimetry assay [36,37]. Since Nrf2 is normally sequestered in the cytoplasm of cells and bound to Keap1, it should be released prior to nuclear translocation and subsequent transactivation. Thus, the effects of **CPUY192018** on the expression of the Nrf2 protein in the HK-2 cells, a line of proximal tubular epithelial cells from normal human kidney, was first evaluated. As shown in Fig. 1A, treatment with **CPUY192018** resulted in a concentration-dependent increase of Nrf2 protein levels. Ubiquitination analysis demonstrated that Nrf2 ubiquitination was decreased by **CPUY192018** (Fig. 1B), which implied that **CPUY192018** likely impairs the ubiquitin-mediated protein degradation system to reduce Nrf2 degradation and thereby elevates the protein levels of Nrf2. To further investigate the effects of **CPUY192018** on Nrf2-ARE activation, we examined the subcellular localization of the Nrf2 protein in HK-2 cells. As expected, higher Nrf2 expression and predominant nuclear localization of Nrf2 were observed in response to **CPUY192018** treatment, which began within 2 h and reached a maximum at 8 h (Fig. 1C). Immunofluorescence staining was further used to assess the relative expression and localization of the Nrf2 protein in HK-2 cells in response to **CPUY192018**. Consistent with the western blotting result, cells exposed to **CPUY192018** (10 μM) showed increased Nrf2 protein staining (green staining) and nuclear accumulation (colocalization with blue DAPI staining) compared to control cells (Fig. 1D).

To explore the biological relevance of **CPUY192018** on the Nrf2-ARE regulated transcription capacity, a cell-based ARE-luciferase reporter assay was performed. HepG2-ARE-C8 cells that had been stably transfected with a luciferase reporter were treated with various concentrations (0.01, 0.1, 1, 5, 10 and 20 μM) of **CPUY192018** or t-BHQ for the indicated times. **CPUY192018** exhibited a concentration-dependent pattern for ARE inductivity, which was far more efficacious than the classical Nrf2 activator t-BHQ (Fig. 1E). Collectively, these data confirmed that **CPUY192018** was capable of inhibiting Keap1-dependent Nrf2 ubiquitination, leading to an increase of Nrf2 protein and its subsequent translocation to the nucleus, which significantly activated Nrf2-ARE signalling.

### 3.2 CPUY192018 significantly upregulated the Nrf2-ARE controlled cytoprotective genes in HK-2 cells in a Nrf2-dependent manner

**Fig. 2:**
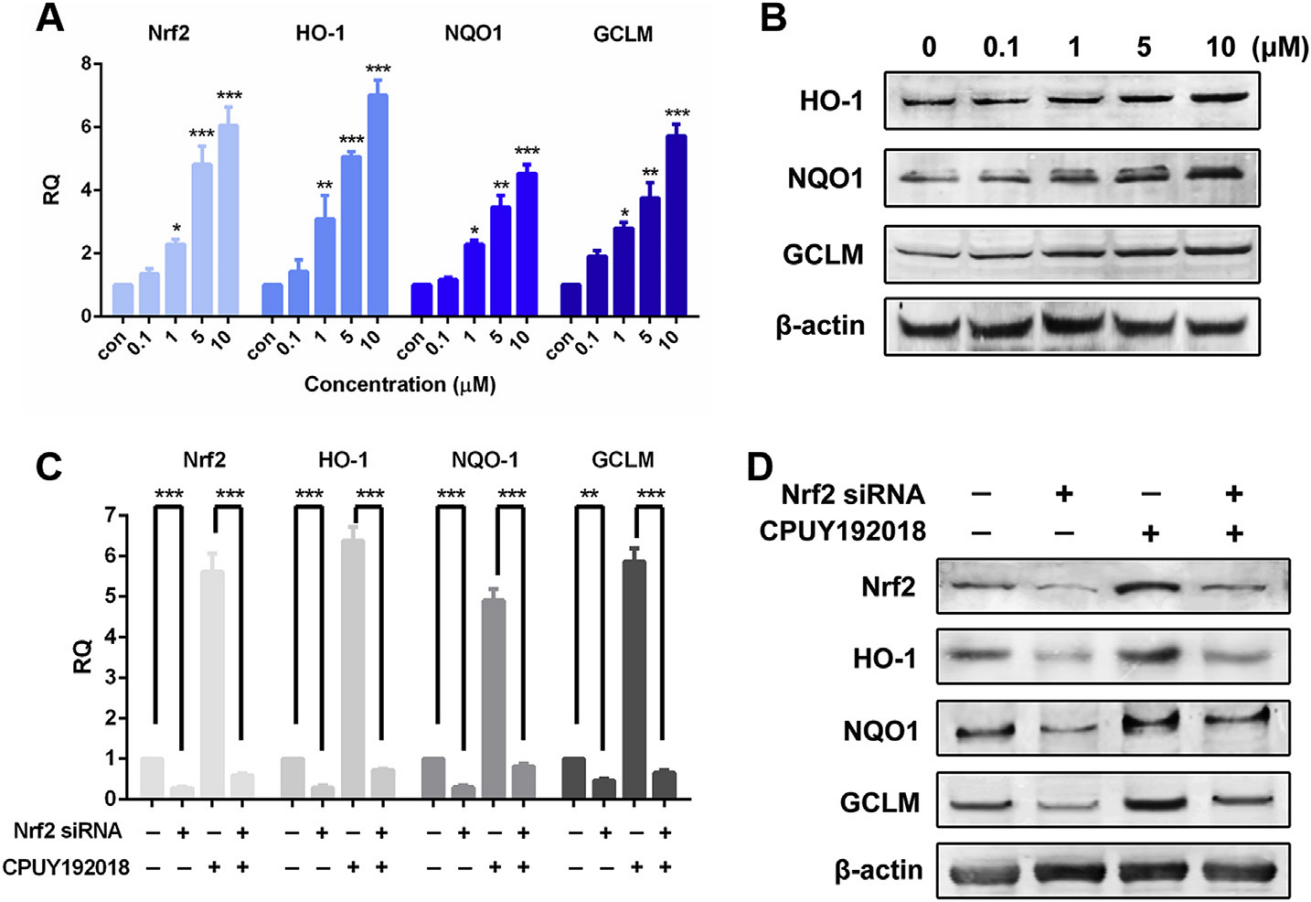
**CPUY192018 significantly upregulated the Nrf2-ARE controlled cytoprotective genes in HK-2 cells in a Nrf2-dependent manner**. (A) Quantitative real-time PCR analysis of Nrf2, HO-1, NQO1 and GCLM in HK-2 cells. The mRNA levels of Nrf2 and the Nrf2-targeted genes were measured at 10 h after treatment of HK-2 cells with various concentrations (0.1, 1, 5, 10 μM) of **CPUY192018**. β-actin was used to normalize the expression of these genes. (B) Western blot analysis of the Nrf2 downstream proteins HO-1, NQO1 and GCLM in HK-2 cells after treatment with various concentrations (0, 0.1, 1, 10 μM) of **CPUY192018** for 8 h. (C) The mRNA expression of Nrf2 and the Nrf2-regulated genes after exposure to Nrf2 siRNA and **CPUY192018**. HK-2 cells were treated with Nrf2 siRNA (50 nM), **CPUY192018** (10 μM), or Nrf2 siRNA (50 nM) plus **CPUY192018** (10 μM). (D) Western blot analysis of Nrf2 and the Nrf2-regulated proteins after exposure to Nrf2 siRNA and **CPUY192018**. HK-2 cells were treated with Nrf2 siRNA (50 nM), **CPUY192018** (10 μM), or Nrf2 siRNA (50 nM) plus **CPUY192018** (10 μM). Additional HK-2 cells were treated with a scrambled duplex for use as a blank control. The values shown are the means ± SEM (n = 3 independent observations). ***P < 0.001, **P < 0.01, and *P < 0.05, one-way ANOVA with Tukey–Kramer posttest.

Increased nuclear levels of Nrf2 and subsequent ARE transactivation provoke gene transcription of detoxifying enzymes which promote glutathione synthesis, detoxify reactive intermediates and metabolites and quench ROS. Examples of Nrf2 cytoprotective target genes include haem oxygenase-1 (HO-1), NAD(P)H:quinone oxidoreductase 1 (NQO1) and glutamate-cysteine ligase modifier subunit (GCLM) [39]. HO-1 is the rate-limiting enzyme in haem catabolism and is induced by a wide variety of oxidative agents. Nrf2-induced HO-1 elevation may therefore function as an endogenous defensive mechanism to attenuate inflammatory events in the kidney [40]. NQO1 is a prototypic phase II detoxifying enzyme that encodes a cytoplasmic 2-electron reductase [41]. The promoter region of the NQO1 gene contains multiple copies of ARE, which is precisely regulated by Nrf2 and therefore contributes to Nrf2 downregulated enzyme induction. Research has described the pivotal role of NQO1 in the protection against the toxic and carcinogenic effects of electrophiles and oxidants [42]. GCLM is a modulatory subunit of glutamate-cysteine ligase (GCL), the rate-limiting enzyme for glutathione (GSH) synthesis [43]. To ascertain the effects of **CPUY192018** on the expression of the Nrf2-ARE-driven genes in HK-2 cells, the mRNA expression profiles of Nrf2 and the three selected genes described above, HO-1, NQO1 and GCLM, were examined by the quantitative real-time PCR. Treatment of the HK-2 cells with **CPUY192018** (0.1–10 μM) induced a concentration-dependent increase in the mRNA levels of Nrf2 and its target genes (Fig. 2A). At the highest concentration of **CPUY192018** (10 μM), the levels of mRNA for Nrf2, HO-1, NQO1 and GCLM were enhanced 6.1-, 7.0-, 4.5- and 5.7-fold, respectively. In addition, increased transcription as a result of **CPUY192018** treatment was accompanied by increased expression of protein levels in HK-2 cells (Fig. 2B).

To determine whether the **CPUY192018** induction of target genes was Nrf2-dependent, HK-2 cells were transfected with a siRNA against human Nrf2, prior to the treatment with **CPUY192018**. As shown in Fig. 2C, knocking down Nrf2 significantly reduced the mRNA levels of Nrf2 and its target genes. As expected, Nrf2 knock down abolished the **CPUY192018**-induced increase of mRNA level of Nrf2 and its downstream genes in HK-2 cells, and the upregulation of the protein level of Nrf2 along with these target proteins was also significantly impaired (Fig. 2D). Altogether, these results demonstrated that **CPUY192018** activated Nrf2-ARE signalling *via* a Nrf2-dependent mechanism in HK-2 cells.

### 3.3 CPUY192018 enhanced the antioxidant capacity of HK-2 cells

**Fig. 3:**
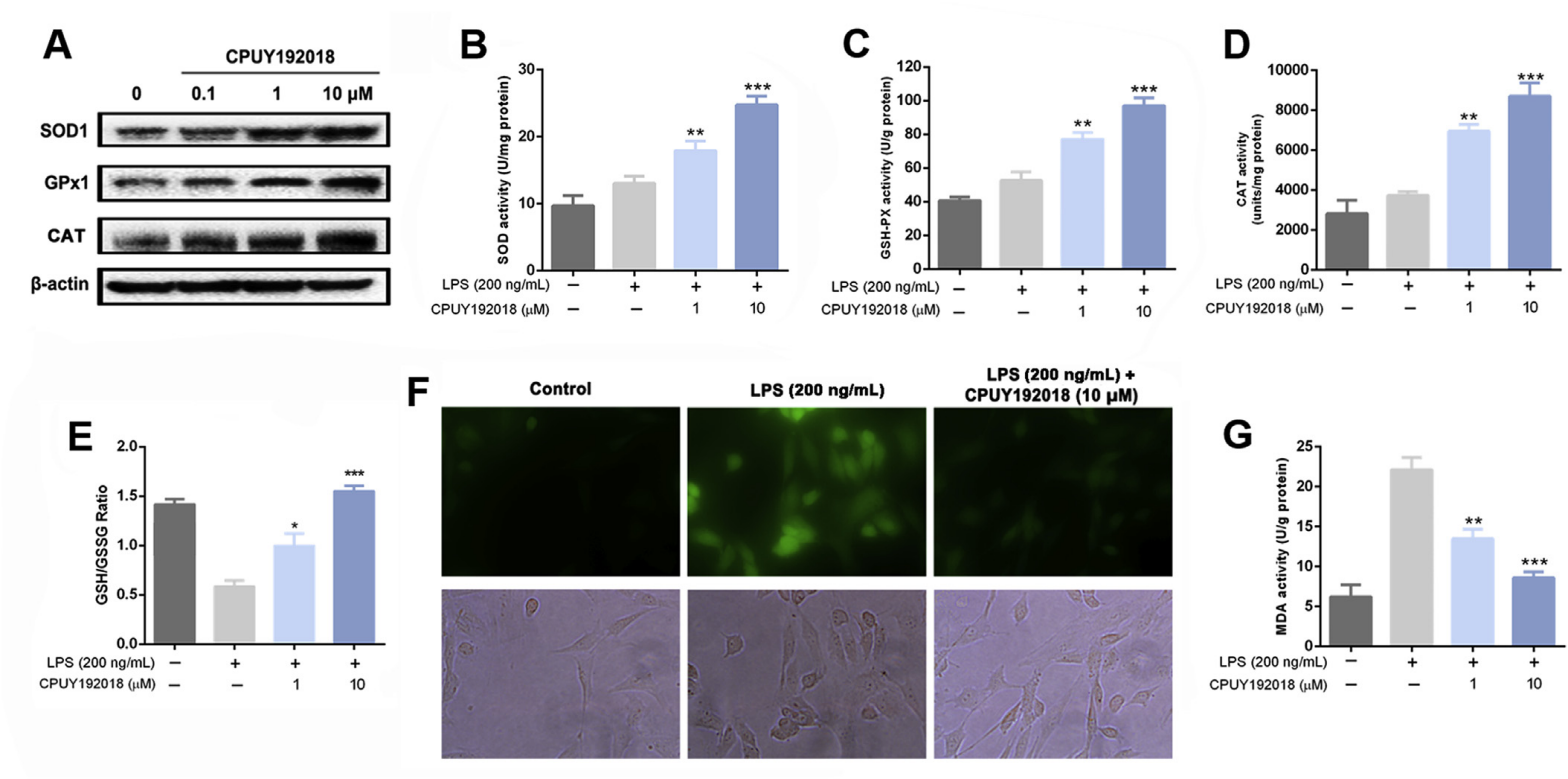
**CPUY192018 enhanced the antioxidant capacity in HK-2 cells**. (A) Western blot analysis of the proteins SOD1 and GPx2 in human HK-2 cells after treatment with various concentrations (0, 0.1, 1, 10 μM) of **CPUY192018** for 10 h. The activities of SOD (B), GSH-Px (C), CAT (D) and MDA (G) were measured in HK-2 cells. (E) The ratio of GSH/GSSG were measured in HK-2 cells. (F) Living Cell Microscopy. HK-2 cells were pretreated with 10 μM **CPUY192018** for 10 h and then exposed to LPS (200 ng/mL) for an additional 6 h. After this treatment, the cells were stained with 10 μM cH_2_DCF-DA for 20 min at 37 °C and living cell fluorescence microscopy was performed. The values shown are the means ± SEM (n = 3 independent observations). ***P < 0.001, **P < 0.01 and *P < 0.05, one-way ANOVA with Tukey–Kramer posttest.

After verifying the cellular Nrf2 activation effects of **CPUY192018**, we then explored whether it could enhance the antioxidant capacity of HK-2 cells against LPS-induced cellular oxidative stress. We first investigated the impact of **CPUY192018** on key antioxidant enzymes, namely, superoxide dismutase (SOD), glutathione peroxidase (GPx) and catalase (CAT). The protein levels of SOD1, GPx2 and CAT in HK-2 cells were first determined by western blotting analysis. HK-2 cells were treated with various concentrations (0, 0.1, 1, 10 μM) of **CPUY192018** for 10 h. Fig. 3A illustrates that **CPUY192018** markedly induced the protein expression of these three antioxidant proteins in HK-2 cells at the concentration of 1 μM. Then, the enzyme activities of SOD, GPx and CAT in HK-2 cells were measured to determine whether **CPUY192018** could enhance reducing metabolic capacity. As shown in Fig. 3B–D, LPS treatment (200 ng/mL) can slightly enhance the activity of SOD, GPx and CAT in HK-2, and pretreatment with **CPUY192018** significantly increased the activities of these three antioxidant enzymes at a low concentration of 1 μM, indicating the enhancement of the antioxidant capacity of HK-2 cells. GSH is an important endogenous antioxidant protein, which can restore cellular homeostasis by preventing damages to important cellular components caused by ROS [44]. The Keap1-Nrf2-ARE signalling dominates the GSH-based antioxidant system [45]. Thus, we further measured the GSH/GSSG ratio in HK-2 cells as an indicator of intracellular antioxidant proteins. As shown in Fig. 3E, treatment with LPS, which can induce the immune response and stress conditions, remarkably reduced the ratio of GSH/GSSG. Pretreatment of **CPUY192018** significantly restored this decline, indicating that **CPUY192018** promoted the synthesis of antioxidant GSH.

Then, we investigated the impact of **CPUY192018** on cellular ROS level in LPS-treated HK-2 cells. Intracellular ROS production was measured by detecting the fluorescence intensity of the oxidation product of the incorporated c-H_2_DCF-DA. An exposure to LPS at a concentration of 200 ng/mL for 6 h resulted in high levels of ROS as shown by examination of the fluorescence image (Fig. 3F, green colour), whereas **CPUY192018** (10 μM) significantly suppressed the production of ROS, confirming that **CPUY192018** can downregulate the ROS level under stressed conditions in HK-2 cells. The reactive species malondialdehyde (MDA) is an endogenous genotoxic product of enzymatic and oxygen radical-induced lipid peroxidation which is commonly used as a biomarker of oxidative stress [46]. LPS (200 ng/mL) exposure resulted in the increase of MDA, while pretreatment of **CPUY192018** effectively antagonized this effect, indicating that **CPUY192018** could relieve the LPS-induced oxidative stress in HK-2 cells (Fig. 3G). Collectively, the enhancement of antioxidant enzymes activity and the increase of the GSH/GSSG ratio confirmed that **CPUY192018** could enhance the antioxidant capacity of HK-2 cells, which reduced the LPS-induced production of ROS and MDA.

### 3.4 CPUY192018 produced cytoprotective effects against the LPS-induced cytotoxicity in HK-2 cells

**Fig. 4:**
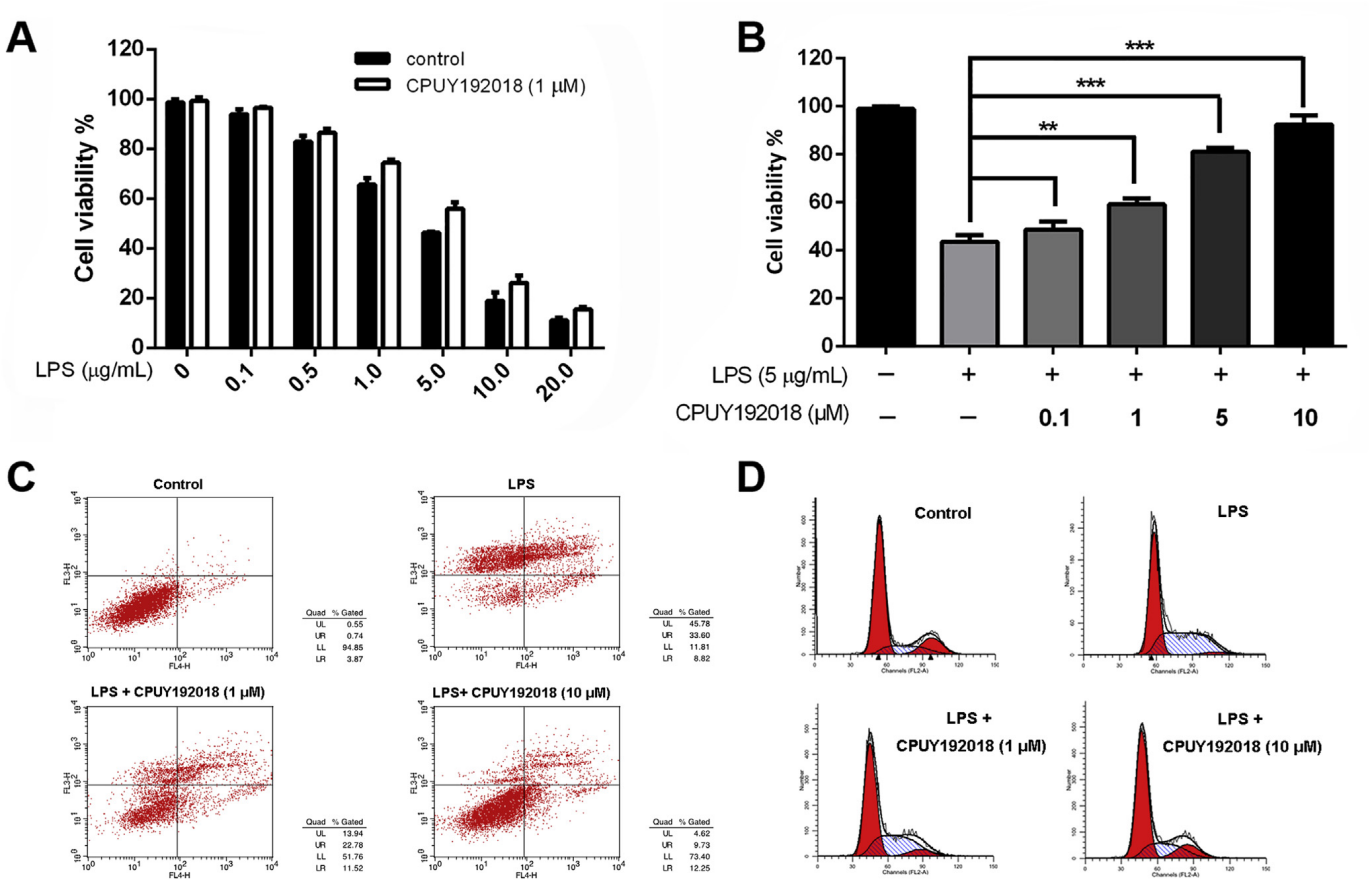
**Effects of CPUY192018 on the LPS-induced injury in HK-2 cells**. (A) Protective effects of **CPUY192018** against LPS-induced cell damage. HK-2 cells were pretreated with 1 μM **CPUY192018** for 10 h then exposed to various concentrations of LPS for an additional 12 h. Cell viability was determined using the MTT assay. The values shown are the means ± SEM (n = 3 independent observations). (B) Concentration-dependent protective effects of **CPUY192018** against the LPS-induced cell damage. HK-2 cells were pretreated with 0.1–10 μM **CPUY192018** for 10 h then exposed to 5 μg/mL LPS for an additional 12 h. The cell viability was determined using the MTT assay. The values shown are the means ± SEM (n = 3 independent observations). (C) Flow cytometry analysis of the apoptotic rate. HK-2 cells were treated with **CPUY192018** for 10 h before being exposed to 5 μg/mL LPS for an additional 8 h. The apoptotic rates were detected by flow cytometry. The statistical analysis of the apoptotic rates is shown in figure. (D) The effect of **CPUY192018** on the cell cycle in HK-2 cells. HK-2 cells were treated with **CPUY192018** for 10 h before being exposed to 5 μg/mL LPS for an additional 8 h. The effects of **CPUY192018** on the cell cycle in HK-2 cells were analyzed by flow cytometry. The statistical analysis of the ratio of HK-2 cells in the G0/G1, S and G2/M phases of the cell cycle are shown in the figure.

Overwhelming evidence indicated that oxidative stress is a major pathogenic factor for the development of renal inflammatory diseases. Therefore, we asked whether **CPUY192018**-induced antioxidant activities could relieve oxidative injury. HK-2 cells were cultured to near confluence, and then cells that were pretreated with or without **CPUY192018** for 10 h were exposed to LPS injury for another 6 h, at which time they were harvested, and their viability was assessed by MTT assay. According to the measurement of cell viability, the number of viable HK-2 cells was significantly decreased after exposure to LPS, compared with the control group. Particularly, when the concentration of LPS was higher than 5 μg/mL, the survival rate of HK-2 cells was less than 50% (Fig. 4A). However, prior treatment with **CPUY192018** for 10 h resulted in substantial concentration-dependent protection against the LPS-induced cytotoxicity, and the survival of HK-2 cells was improved as the concentration of **CPUY192018** increased, with 92% of cells pretreated with 10 μM of **CPUY192018** being viable (Fig. 4B). To further confirm the protective effects of **CPUY192018** against LPS, we also conducted flow cytometry detection to quantify dead cells among the cultured HK-2 cells. As shown in Fig. 4C, exposure to 5 μg/mL LPS caused the apoptosis rate to increase to approximately 42% in the HK-2 cells. Pretreatment with 10 μM **CPUY192018** significantly decreased the LPS-induced apoptosis level to approximately 21%. Then, we investigated the effect of **CPUY192018** on the cell cycle of HK-2 cells. As shown in Fig. 4D, LPS caused partial cell cycle arrest at the S checkpoint in HK-2 cells, while pretreatment with **CPUY192018** effectively restored it to the normal level. Taken together, these results suggested that **CPUY192018** protected HK-2 cells from the LPS-induced cytotoxicity.

### 3.5 CPUY192018 reduced inflammatory factors production and inhibited NF-κB activation induced by LPS in HK-2 cells

**Fig. 5:**
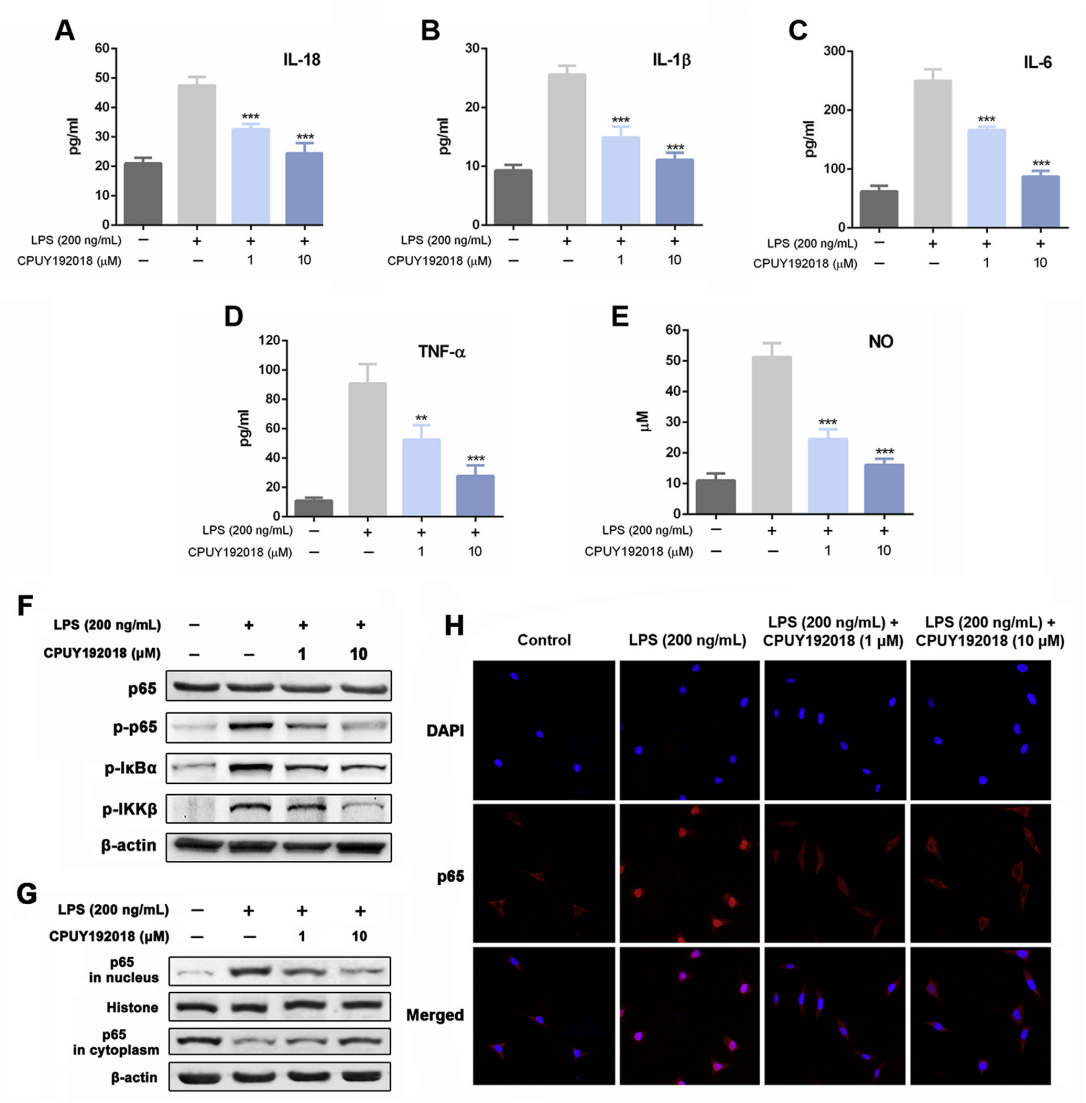
**CPUY192018 reduced inflammatory factors production and inhibited NF-κB activation induced by LPS in HK-2 cells**. (A–E) Quantification of the inflammatory factors IL-18 (A), IL-1β (B), IL-6 (C), TNF-α (D) and NO (E) in HK-2 cells. HK-2 cells were pretreated with **CPUY192018** for 10 h and then exposed to 200 ng/mL LPS for an additional 6 h. The results are expressed as the means ± SEM (n = 3 independent observations). *p < 0.05, **p < 0.01 and ***p < 0.001, one-way ANOVA with Tukey–Kramer posttest. (F) Western blot analysis of the proteins p65, p-p65, p-IκBα and p-IKKβ in human HK-2 cells. HK-2 cells were pretreated with **CPUY192018** for 10 h and then exposed to 200 ng/mL LPS for an additional 6 h. (G) Effect of **CPUY192018** on the nuclear translocation of the NF-κB p65 protein. Nuclear and cytoplasmic cell extracts were prepared from HK-2 cells and subjected to Western blot analysis. Histone and β-actin served as markers for nuclear and cytosolic Nrf2 proteins, respectively. (H) Immunofluorescence staining of NF-κB p65 in HK-2 cells. The bars indicate the magnification (10 μm).

The anti-inflammatory effects of **CPUY192018** were then evaluated in HK-2 cells. As shown in Fig. 5A–E, HK-2 cells that were exposed to LPS showed a remarkable increase in inflammatory factors, including IL-18, IL-1β, IL-6, TNF-α and NO, while pretreatment with both low and high concentrations of **CPUY192018** significantly inhibited the production of these inflammatory factors, which strongly supported the anti-inflammation effects of **CPUY192018** on LPS-treated HK-2 cells.

Considering the production of these pro-inflammatory factors are closely related with redox-regulated transcription factor nuclear factor-kappa B (NF-κB) which can be activated by LPS to induce the inflammatory response, the influence of **CPUY192018** on the NF-κB signal pathway was investigated. LPS (200 ng/mL) treatment significantly elevated the phosphorylation levels of IκB kinase β (IKKβ), IκBα and NF-κB p65 compared to the control group (Fig. 5F), which indicated that the NF-κB activity was activated by LPS in HK-2 cells in a canonical way. Treatment of **CPUY192018** greatly inhibited the phosphorylation of these proteins. We then studied the subcellular translocation of NF-κB and found that NF-κB p65 levels in nuclear fractions were greatly enhanced and the levels in cytoplasmic fractions were greatly decreased in LPS-treated HK-2 cells (Fig. 5G). From the cytosol to the nucleus, the translocation of NF-κB p65 was remarkably inhibited by **CPUY192018**. Furthermore, immunofluorescence analysis was carried out using a primary antibody against the p65 subunit of NF-κB and the nuclei dye DAPI (Fig. 5H). Stimulation with LPS (200 ng/mL) for 6 h induced notable nuclear translocation of p65 in comparison with the control group, and this effect was markedly suppressed by pretreatment with **CPUY192018**. These data suggested that **CPUY192018** could inhibit the LPS-induced NF-κB activation in HK-2 cells, which took part in the anti-inflammatory response of **CPUY192018**.

### 3.6 CPUY192018 ameliorated the pathological symptoms in mice model of the LPS-induced chronic renal inflammation

**Fig. 6:**
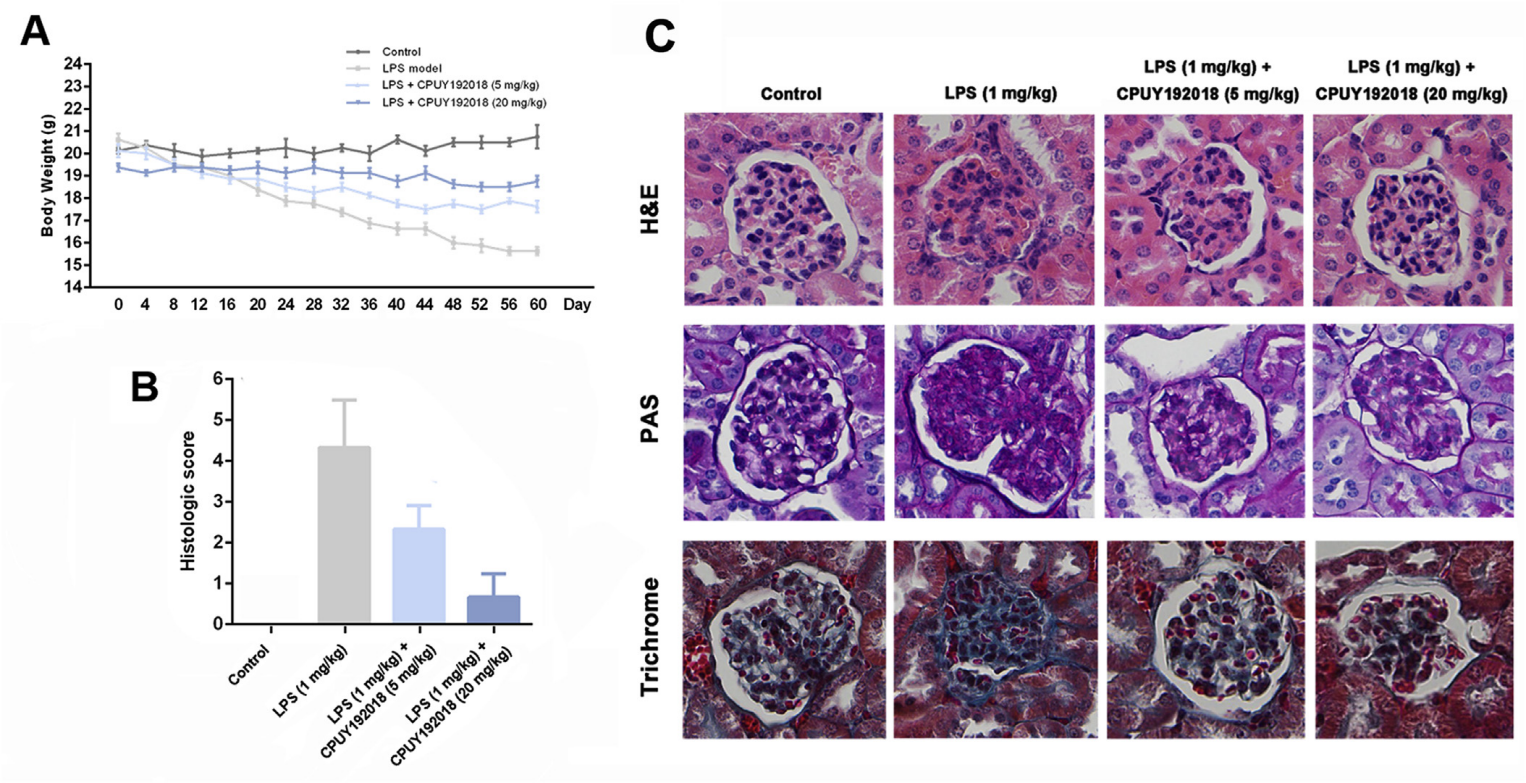
**CPUY192018 ameliorated the pathological symptoms in the LPS-induced chronic renal inflammation in mice**. The animals were randomly assigned to one of the four treatment groups: Control group; LPS model group (the mice received 1 mg/kg LPS through i.p. Injection); LPS + **CPUY192018** (5 mg/kg) group (the mice received 1 mg/kg LPS together with 5 mg/kg **CPUY192018** through i.p. Injection); and LPS + **CPUY192018** (20 mg/kg) group (the mice received 1 mg/kg LPS together with 20 mg/kg **CPUY192018** through i.p. Injection). (A) Gradual changes in body weight during the LPS administration in mice. (B) Histologic inflammatory score. The results are expressed as the means ± SEM (n = 8, in each group). *p < 0.05, **p < 0.01 and ***p < 0.001, one-way ANOVA with Tukey–Kramer posttest. (C) Representative histological images of kidney sections stained with H&E, PAS and Trichrome.

The cytoprotective and anti-inflammatory effects of **CPUY192018** in HK-2 cells inspired further evaluation of the *in vivo* therapeutic potential in the LPS-induced chronic renal inflammation model. Mice in the control group showed a stable increase in their body weight. However, decreased body weight was observed in the LPS-treated mice compared with the control group (Fig. 6A). A grading system was introduced to compare the severity of the histologic signs of the LPS-induced chronic renal inflammation [47]. Likewise, mice treated with LPS exhibited high histological disease scores compared with that of the control group (Fig. 6B). However, these injuries were alleviated by the **CPUY192018** administration as reflected by attenuation of the body weight loss and reduction in the histological disease scores from 4.33 to 0.67.

Representative photomicrographs of kidney histology are presented in Fig. 6C. **CPUY192018** treatment significantly ameliorated the pathological alterations of the glomerulus as indicated by histological examination. Glomerular lesions, including K–W (Kimmelstiel Wilson) nodules, were observed in haematoxylin and eosin (H&E)-stained kidney sections from mice treated with LPS (Fig. 6C, H&E panel). Treatment with **CPUY192018** effectively restored the normal morphology of glomeruli and resulted in slight swelling of the tubular epithelium. Glycogen deposition in the glomeruli was measured by periodic acid Schiff (PAS) staining to evaluate the severity of glomerulosclerosis. PAS staining showed that LPS induced obvious glomerulosclerosis, which was significantly improved in **CPUY192018**-treated mice (Fig. 6C, PAS panel). Moreover, the tissue section was stained with Masson's trichrome for detection of extracellular matrix (ECM) deposition contents, which is another hallmark of glomerulosclerosis. As shown in Fig. 6C, LPS treatment resulted in significant collagen accumulation inside glomeruli or in the periglomerular area, which was effectively reduced in **CPUY192018**-treated groups (Fig. 6C, trichrome panel). Taken together, these results clearly demonstrated the ability of **CPUY192018** to attenuate the LPS-induced pathological alterations both in renal function and structure during the progression of chronic renal inflammation.

### 3.7 CPUY192018-induced activation of the Nrf2 pathway confers protection against renal oxidative damage

**Fig. 7:**
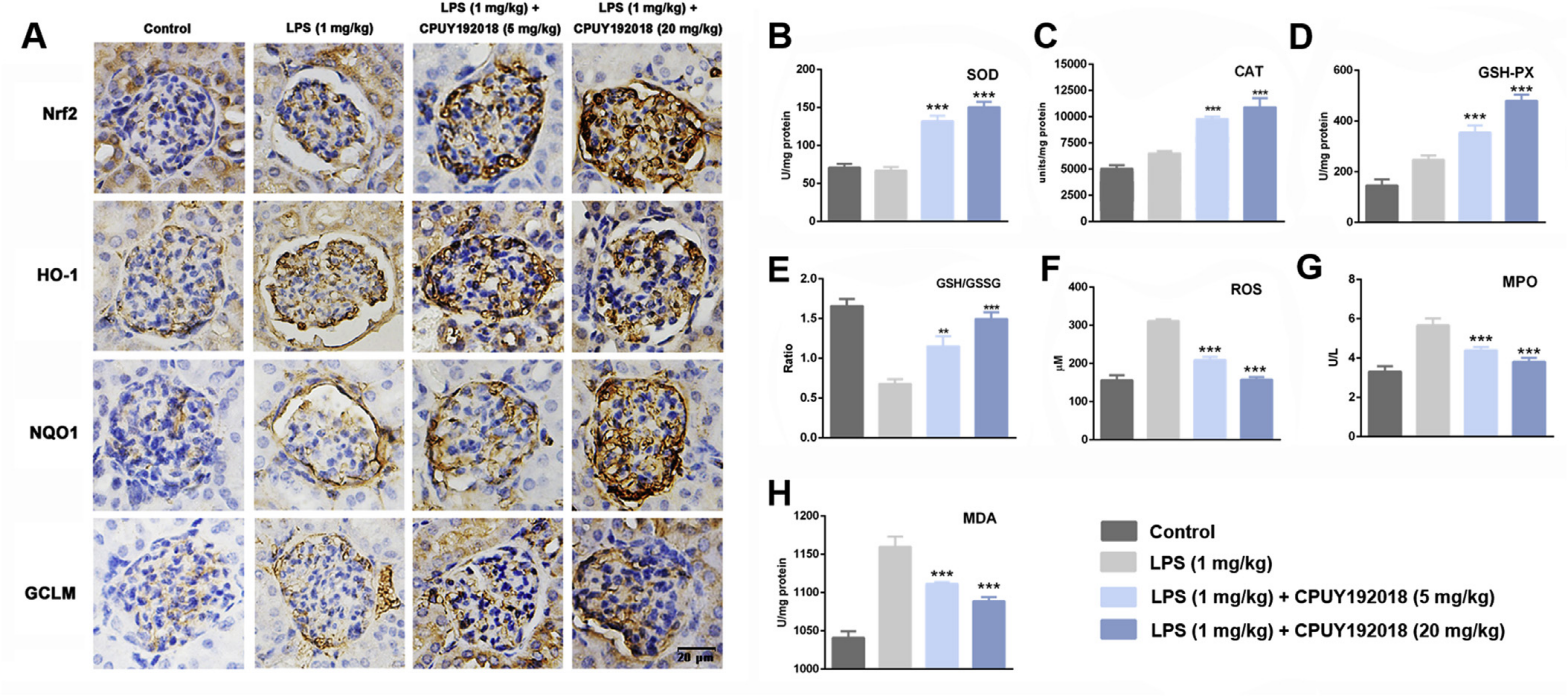
**CPUY192018-induced activation of the Nrf2 pathway confers protection against renal oxidative damage**. (A) Immunohistochemical detection of Nrf2 together with its target proteins HO-1, NQO1 and GCLM levels in the kidney sections from mice. The bars indicate the magnification (20 μm). The activities of SOD (B), CAT (C), GPX (D), MPO (G) and MDA (H), the GSH/GSSG ratio (E) and the ROS level (F) were measured in the mouse kidneys. The results are expressed as the means ± SEM (n = 8, in each group). *p < 0.05, **p < 0.01 and ***p < 0.001, one-way ANOVA with Tukey–Kramer posttest.

Because **CPUY192018** protected the HK-2 cells against the LPS-induced cell damage by activating Nrf2-dependent cytoprotective enzymes, we then evaluated whether **CPUY192018** could activate Nrf2 and downstream enzymes in renal tissues as well. As shown in Fig. 7A, expression of Nrf2 and its downstream targets HO-1, NQO1 and GCLM was slightly increased after LPS injection, indicating induction of the Nrf2 pathway by renal oxidative stress. As expected, administration of **CPUY192018** markedly upregulated the protein levels of Nrf2 and its targets, which further confirmed the *in vivo* Nrf2-ARE activation effects of **CPUY192018** (Fig. 7A). Consistent with the results of the *in vitro* studies, treatment with **CPUY192018** also clearly upregulated the endogenous antioxidant enzymes including SOD, CAT and GPx (Fig. 7B–D). Then, we measured the GSH/GSSG ratio in the kidney as an indicator of the GSH-based antioxidant system. As shown in Fig. 7E, the concentration of GSH was markedly reduced and that of oxidized glutathione (GSSG) was increased. Therefore, the GSH-to-GSSG ratio was markedly diminished in the LPS model animals, while treatment with **CPUY192018** significantly increased this ratio. Evidence suggested that the LPS-induced generation of ROS contributes substantially to inflammation as well as the oxidative tissue injury. We then examined whether **CPUY192018** affected the ROS level that was elevated by LPS in mouse kidney. As illustrated in Fig. 7F, mice treated with LPS exhibited a remarkably high level of intrarenal ROS. On the other hand, such an effect was effectively reversed by co-administration with **CPUY192018**. Myeloperoxidase (MPO) is an important pathogenic factor in glomerular and tubulointerstitial diseases [48,49]. We therefore further measured MPO activity in the LPS-induced chronic renal inflammation mice model. The mice given LPS alone showed an obvious increase in MPO activity compared to the control group. Treatment with **CPUY192018** at 20 mg/kg/day markedly reduced MPO activity to a level nearly equal to that of the control group (Fig. 7G). The MDA level in the kidney tissue was also measured to evaluate oxidative assault during the progress of chronic inflammatory conditions. LPS treatment markedly increased MDA levels (Fig. 7H), and pretreatment with **CPUY192018** significantly alleviated these effects. Collectively, these results indicated that **CPUY192018** significantly activated the Nrf2 regulated antioxidant system in kidney tissue and effectively antagonized LPS-induced oxidative stress conditions.

### 3.8 CPUY192018 relieved the LPS-induced inflammatory conditions in the kidney

**Fig. 8:**
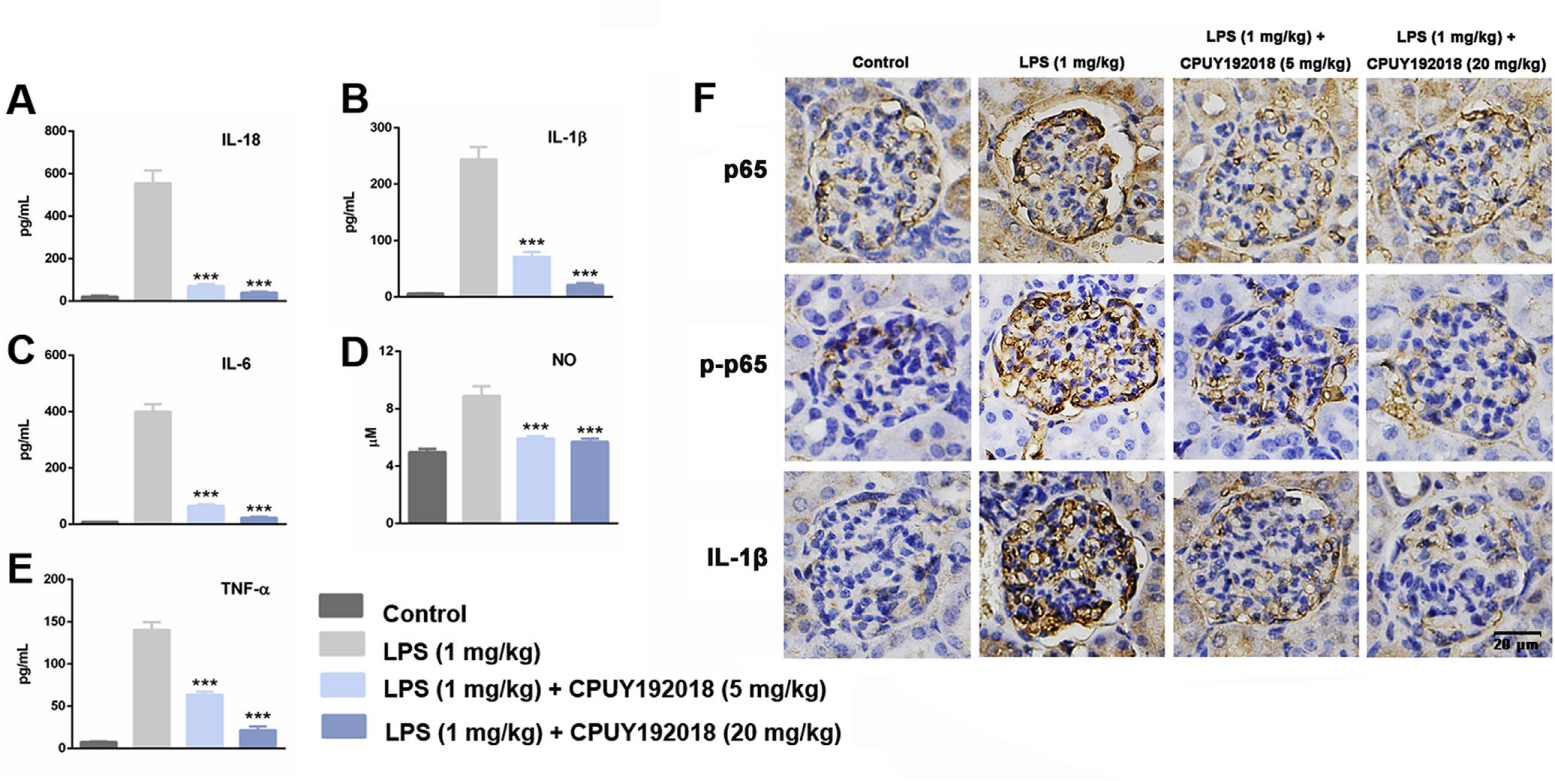
**CPUY192018 relieved the LPS-induced inflammatory conditions in the kidney**. (A–E) The levels of serum IL-18, IL-1β, IL-6, TNF-α and NO activity. The results are expressed as the means ± SEM (n = 8, in each group). *p < 0.05, **p < 0.01 and ***p < 0.001, one-way ANOVA with Tukey–Kramer posttest. (F) Immunohistochemical detection of p65, p-p65 and IL-1β protein levels in the kidney sections from mice. The bars indicate the magnification (20 μm).

A complex array of inflammatory signalling processes impairs kidney function and leads to the recruitment of inflammatory cells to the site of injury. Therefore, we further assessed several inflammatory markers to evaluate the effects of **CPUY192018** on LPS-induced renal inflammatory conditions. After 8 weeks administration of LPS, the levels of serum IL-18, IL-1β, IL-6, TNF-α and NO were remarkably increased by LPS-treatment, and administration of **CPUY192018** significantly reduced the amount of these inflammatory factors (Fig. 8A–E). These factors are closely related to the activation of redox-sensitive NF-κB. We further asked whether **CPUY192018** inhibits NF-κB activation in LPS-treated kidney tissue. As shown in Fig. 8F, compared with the control group, LPS treated mice showed significantly increased renal phosphorylation levels of NF-κB p65 by IHC, indicating activation of NF-κB in kidney. However, this effect was significantly decreased in **CPUY192018** treated mice. These phenomena were consistent with the results in the culture system of HK-2 cells (Fig. 6). Consistent with the ELISA result, the marked reduction of IL-1β levels was also observed by IHC of kidney tissue. These results showed that **CPUY192018** significantly suppressed the inflammatory conditions and effectively inhibited the LPS-induced NF-κB activation in the kidneys.

## 4 Discussion

CKD is an increasing public health issue, of which the prevalence is estimated to be 8–16% worldwide [50]. While CKD is being increasingly diagnosed worldwide, therapies such as dialysis or transplantation are either too costly or ineffective. Currently, there is no single treatment to improve kidney function in CKD, and the unrelenting decline in kidney function means that the condition progresses to overt kidney failure. In this way, finding an effective treatment remains an urgent priority.

Oxidative stress and its constant companion inflammation are common features and major mediators of CKD initiation and progression and the associated complications [51]. Thus, factors that control this pathway may be attractive drug targets. Increased production of ROS in CKD is companied by an impaired endogenous antioxidant defence system in the kidney. Previous pharmacological studies have focused on preventing ROS generation and supplying or boosting individual cellular antioxidants and ROS scavengers; however, these methods have been limited by various complicating factors, including dosage and *in vivo* stability, and better targets remain to be investigated. Nrf2 is a cytoprotective transcription factor and has a central role in the basal activity and coordinated induction of target gene products, including antioxidant enzymes (that is, SOD isoforms, GPx, CAT and HO-1), the key enzymes responsible for glutathione synthesis (that is, glutamate–cysteine ligase catalytic (GCLC) and GCLM), and the major detoxifying enzyme NQO-1 [16], which play important roles in the cellular defence system, especially in oxidative stress modulation. Given the role of oxidative stress and inflammation in the pathogenesis of CKD, Nrf2-targeted strategies may hold promise for prevention of CKD progress in humans.

The Keap1-Nrf2 system is one of the most promising therapeutic targets for inflammatory kidney disease. Directly disrupting the Keap1-Nrf2 complex can activate the protein levels of Nrf2 and thereby provide a cell protection mechanism against oxidative assault when endogenous stress defence mechanisms are imbalanced, which could be part of critical therapies for kidney diseases. Moreover, compared with electrophilic Nrf2 activators, directly disrupting Keap1-Nrf2 interactions using small molecules is a more selective way to enhance Nrf2 activity. These agents can avoid the potential risk of unselected modification of the cysteine residue by electrophilic Nrf2 activators. In this study, **CPUY192018**, with extraordinary Keap1-Nrf2 PPI inhibition potency, was used as a small-molecule probe to explore the effects of the specific activation of the Keap1-Nrf2-ARE system on LPS-induced chronic renal inflammation.

We first confirmed that **CPUY192018** boosted levels of Nrf2 protein and the expression of downstream ARE-driven genes, including HO-1, NQO1 and GCLM in HK-2 cells. Then, we proved that activation of Nrf2 by **CPUY192018** significantly enhanced the antioxidant capacity of HK-2 cells on behalf of the enhancing activity of key antioxidant enzymes including SOD, CAT and GPx and the upregulation of the GSH/GSSG ratio. SOD gives protection by directly scavenging superoxide radicals and hydrogen peroxide (H_2_O_2_), converting them to less reactive species [52]. SOD catalyses the dismutation of the superoxide radical (•O_2_) to H_2_O_2_, which is one of the major enzymes that protect cells from ROS [53]. GPx is a family of selenoproteins that are responsible for the conversion of H_2_O_2_ and other organic peroxides, to water and oxygen [54]. CAT is a common antioxidant enzyme present abundantly in liver, lungs, and kidneys [55]. CAT plays an important role in protecting organisms against oxidative damage caused by ROS by degrading hydrogen peroxide [56]. The role of catalase in defending cells and tissues against oxidative stress has been studied extensively [57]. The enhanced activity of these enzymes confirmed that **CPUY192018** can strengthen the antioxidant system in HK-2 cells, thus **CPUY192018** treatment reduced the low dose LPS-induced oxidative injury proven by downregulation of ROS and MDA levels and high dose LPS-induced cytotoxicity reflected by the elevation of cell survival and normalization of the cell cycle. These results strongly indicated that **CPUY192018** may protect HK-2 cells from the LPS-induced injury *via* the antioxidative pathway.

Then we evaluated the anti-inflammatory effects of **CPUY192018** on HK-2 cells. Treating HK-2 cells with LPS significantly induced the production of inflammatory factors, including IL-18, IL-1β, IL-6, TNF-α and NO, and addition of **CPUY192018** suppressed this inflammatory response. It is noteworthy that these inflammatory factors are all closely related to NF-κB, a key regulator of inflammatory response. The NF-κB pathway can be activated by LPS, and the activation of NF-κB can induce the transcription of a vast range of genes, including inflammatory cytokines such as TNF-α, IL-1β, IL-2, IL-6, IL-12, as well as some leukocyte adhesion molecules [58]. It is not surprising that **CPUY192018** can significantly inhibit LPS-induced NF-κB activation. It is well known that NF-κB is a redox sensitive transcription factor and the reduced microenvironment can inhibit the NF-κB activation, especially for LPS-mediated canonical activation. Considering that **CPUY192018** can significantly downregulate the ROS level, it is reasonable that **CPUY192018** can reduce the production of NF-κB related inflammatory factors. Noteworthily, the important role of Nrf2 in reducing inflammation has been intensively linked to its ability to antagonize NF-κB [59]. The Nrf2 and NF-κB pathways are proposed to inhibit each other at their transcription level *via* PPIs or through secondary messenger effects. The Nrf2 pathway inhibits NF-κB mediated transcription by preventing the degradation of IκBα. Similarly, NF-κB mediated transcription reduces Nrf2 activation by reducing the ARE gene transcription and decreases free CREB binding protein (CBP) by competing with Nrf2 for the CH1-KIX domain of CBP [60]. NF-κB also enhances the recruitment of histone deacetylase3 (HDAC3) to the ARE region by binding to Mafk, therefore interfering with the transcriptional facilitation of Nrf2 [61]. In addition, activation of Nrf2 increases antioxidant defences and HO-1 expression, which efficiently neutralizes ROS and detoxifies toxic chemicals and therefore inhibits ROS-mediated NF-κB activation [62]. Nrf2 also increases intracellular GSH levels and GSH-dependent enzymes, favouring a reducing environment and hence, inhibiting NF-κB [63]. Our study proved that the Keap1-Nrf2 PPI inhibitor **CPUY192018** could suppress the NF-κB signal pathway through the inhibition of nuclear translocation by regulating the phosphorylation of IκBα, IKKβ and p65 and hindering the production of proinflammatory cytokines.

Based on the *in vitro* results, we further evaluated the *in vivo* effects of **CPUY192018** using an LPS-induced chronic renal inflammation model. Kidneys from mice that underwent the LPS administration showed characteristic morphological changes, such as extensive tubular necrosis, tubular dilatation, and loss of brush border. Conversely, pretreatment with **CPUY192018** reduced the amount of renal dysfunction that was induced by LPS treatment. The histopathologic scores supported the histologic findings, and **CPUY192018** treatment reduced the score significantly. These favorable effects of **CPUY192018** resulted in the maintenance of normal body weight following LPS injury. Treatment with **CPUY192018** also preserved the normal morphology of the kidney well. With regards to the kidney severity, further assessment of the antioxidant activity and ROS generation in the kidney tissue also showed that **CPUY192018** can protect against LPS-induced injury by promoting the expression of the classic antioxidant enzymes SOD, CAT and GSH-Px and the clearance of ROS. **CPUY192018** significantly attenuated LPS-induced inflammatory conditions as indicated by reduced levels of inflammatory markers including IL-18, IL-1β, IL-6, NO and TNF-α in the kidney tissue. Further immunohistochemical analysis demonstrated that administration of **CPUY192018** markedly decreased the protein level of phosphor-p65 in mouse kidneys, which confirmed the *in vivo* inhibition of the NF-κB signalling.

**Fig. 9:**
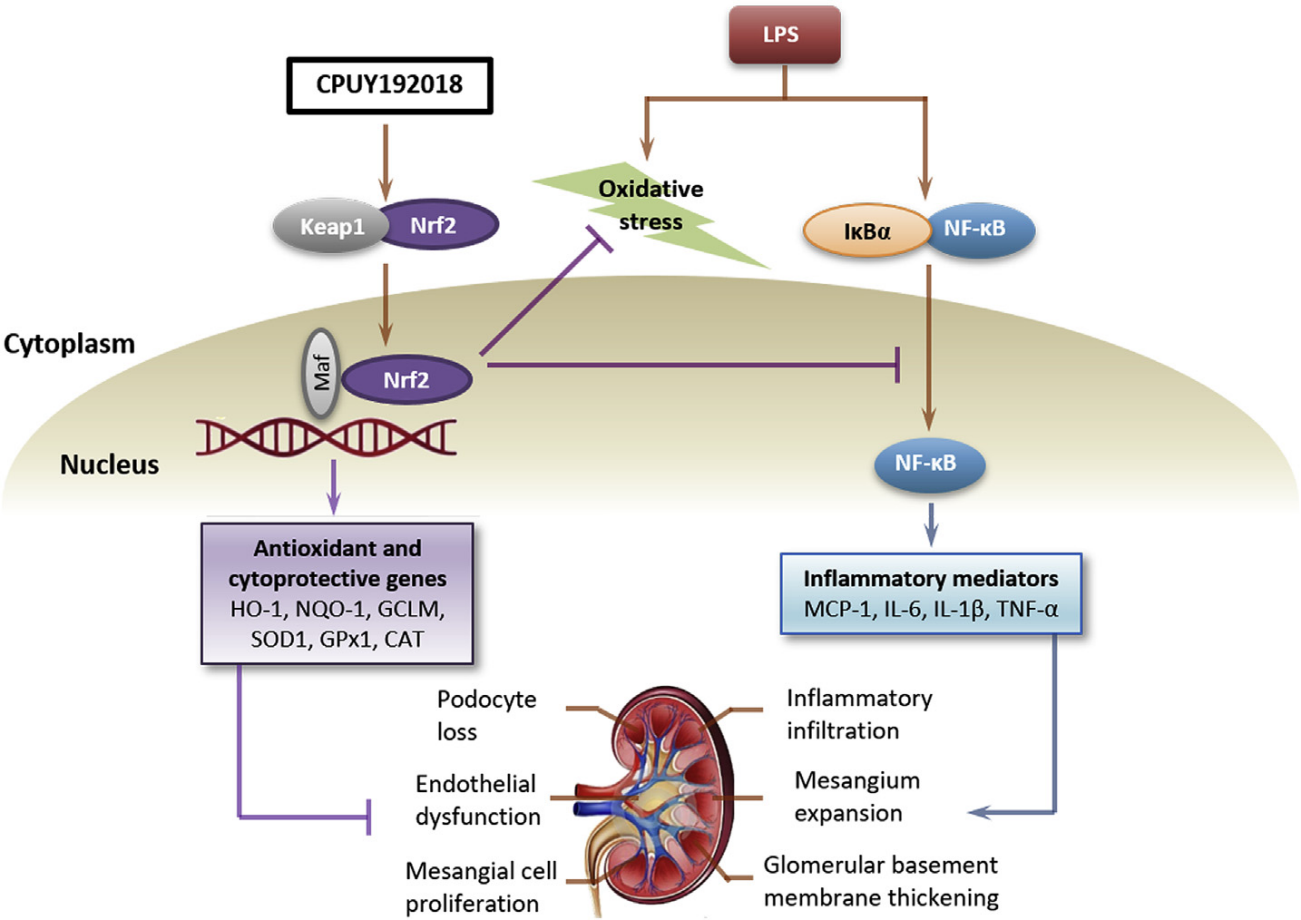
CPUY192018 activates Nrf2-ARE signalling and inhibits inflammatory pathways that lead to kidney dysfunction.

Taken together, as shown in Fig. 9, our identification of **CPUY192018** as a potent Keap1-Nrf2 PPI inhibitor, which activates Nrf2-dependent antioxidative pathways and inhibits NF-κB involved inflammatory response to protect kidney from the LPS-induced chronic renal inflammation both in HK-2 cells and *in vivo*, unveils therapeutic opportunities of inflammatory kidney diseases therapies.

## Conflicts of interest

All authors have given approval to the final version of the manuscript.

## Appendix A Supplementary data

The Supporting Information containing the synthetic route of **CPUY192018** and the cytotoxicity of **CPUY192018** against HK-2 cells is available free of charge on the website.

## References

[1] Tebay L.E., Robertson H., Durant S.T., Vitale S.R., Penning T.M., Dinkova-Kostova A.T., Hayes J.D. (2015). Mechanisms of activation of the transcription factor Nrf2 by redox stressors, nutrient cues, and energy status and the pathways through which it attenuates degenerative disease. Free Radic. Biol. Med..

[2] Baird L., Dinkova-Kostova A.T. (2011). The cytoprotective role of the Keap1-Nrf2 pathway. Arch. Toxicol..

[3] Lu M.-C., Ji J.-A., Jiang Z.-Y., You Q.-D. (2016). The keap1–nrf2–ARE pathway as a potential preventive and therapeutic target: an update. Med. Res. Rev..

[4] Arlt V.M., Stiborova M., Henderson C.J., Osborne M.R., Bieler C.A., Frei E., Martinek V., Sopko B., Wolf C.R., Schmeiser H.H., Phillips D.H. (2005). Environmental pollutant and potent mutagen 3-nitrobenzanthrone forms DNA adducts after reduction by NAD(P)H:quinone oxidoreductase and conjugation by acetyltransferases and sulfotransferases in human hepatic cytosols. Cancer Res..

[5] Keleku-Lukwete N., Suzuki M., Yamamoto M. (2018). An overview of the advantages of KEAP1-NRF2 system Activation during inflammatory disease treatment. Antioxidants Redox Signal..

[6] Tonelli C., Chio I.I.C., Tuveson D.A. (2018). Transcriptional regulation by Nrf2. Antioxidants Redox Signal..

[7] Cuadrado A., Manda G., Hassan A., Alcaraz M.J., Barbas C., Daiber A., Ghezzi P., Leon R., Lopez M.G., Oliva B., Pajares M., Rojo A.I., Robledinos-Anton N., Valverde A.M., Guney E., Schmidt H. (2018). Transcription factor NRF2 as a therapeutic target for chronic diseases: a systems medicine approach. Pharmacol. Rev..

[8] Nezu M., Suzuki N., Yamamoto M. (2017). Targeting the KEAP1-NRF2 system to prevent kidney disease progression. Am. J. Nephrol..

[9] Cuadrado A., Rojo A.I., Wells G., Hayes J.D., Cousin S.P., Rumsey W.L., Attucks O.C., Franklin S., Levonen A.L., Kensler T.W., Dinkova-Kostova A.T. (2019). Therapeutic targeting of the NRF2 and KEAP1 partnership in chronic diseases. Nat. Rev. Drug Discov..

[10] Liu M., Grigoryev D.N., Crow M.T., Haas M., Yamamoto M., Reddy S.P., Rabb H. (2009). Transcription factor Nrf2 is protective during ischemic and nephrotoxic acute kidney injury in mice. Kidney Int..

[11] Liu M., Reddy N.M., Higbee E.M., Potteti H.R., Noel S., Racusen L., Kensler T.W., Sporn M.B., Reddy S.P., Rabb H. (2014). The Nrf2 triterpenoid activator, CDDO-imidazolide, protects kidneys from ischemia-reperfusion injury in mice. Kidney Int..

[12] Yoh K., Hirayama A., Ishizaki K., Yamada A., Takeuchi M., Yamagishi S., Morito N., Nakano T., Ojima M., Shimohata H., Itoh K., Takahashi S., Yamamoto M. (2008). Hyperglycemia induces oxidative and nitrosative stress and increases renal functional impairment in Nrf2-deficient mice. Genes Cells.

[13] Ratliff B.B., Abdulmahdi W., Pawar R., Wolin M.S. (2016). Oxidant mechanisms in renal injury and disease. Antioxidants Redox Signal..

[14] Frijhoff J., Winyard P.G., Zarkovic N., Davies S.S., Stocker R., Cheng D., Knight A.R., Taylor E.L., Oettrich J., Ruskovska T., Gasparovic A.C., Cuadrado A., Weber D., Poulsen H.E., Grune T., Schmidt H.H., Ghezzi P. (2015). Clinical relevance of biomarkers of oxidative stress. Antioxidants Redox Signal..

[15] Jha J.C., Banal C., Chow B.S., Cooper M.E., Jandeleit-Dahm K. (2016). Diabetes and kidney disease: role of oxidative stress. Antioxidants Redox Signal..

[16] Ruiz S., Pergola P.E., Zager R.A., Vaziri N.D. (2013). Targeting the transcription factor Nrf2 to ameliorate oxidative stress and inflammation in chronic kidney disease. Kidney Int..

[17] Itoh K., Mimura J., Yamamoto M. (2010). Discovery of the negative regulator of Nrf2, Keap1: a historical overview. Antioxidants Redox Signal..

[18] Sihvola V., Levonen A.L. (2017). Keap1 as the redox sensor of the antioxidant response. Arch. Biochem. Biophys..

[19] Canning P., Sorrell F.J., Bullock A.N. (2015). Structural basis of Keap1 interactions with Nrf2. Free Radic. Biol. Med..

[20] Kansanen E., Kuosmanen S.M., Leinonen H., Levonen A.L. (2013). The Keap1-Nrf2 pathway: mechanisms of activation and dysregulation in cancer. Redox Biol..

[21] Baird L., Lleres D., Swift S., Dinkova-Kostova A.T. (2013). Regulatory flexibility in the Nrf2-mediated stress response is conferred by conformational cycling of the Keap1-Nrf2 protein complex. Proc. Natl. Acad. Sci. U.S.A..

[22] Baird L., Swift S., Lleres D., Dinkova-Kostova A.T. (2014). Monitoring Keap1-Nrf2 interactions in single live cells. Biotechnol. Adv..

[23] Kern J.T., Hannink M., Hess J.F. (2007). Disruption of the Keap1-containing ubiquitination complex as an antioxidant therapy. Curr. Top. Med. Chem..

[24] Rojo de la Vega M., Dodson M., Chapman E., Zhang D.D. (2016). NRF2-targeted therapeutics: New targets and modes of NRF2 regulation. Curr. Opin. Toxicol..

[25] Dinkova-Kostova A.T., Kostov R.V., Canning P. (2017). Keap1, the cysteine-based mammalian intracellular sensor for electrophiles and oxidants. Arch. Biochem. Biophys..

[26] Suzuki T., Yamamoto M. (2017). Stress-sensing mechanisms and the physiological roles of the Keap1-Nrf2 system during cellular stress. J. Biol. Chem..

[27] Yamamoto M., Kensler T.W., Motohashi H. (2018). The KEAP1-NRF2 system: a thiol-based sensor-effector apparatus for maintaining redox homeostasis. Physiol. Rev..

[28] Wang Y.Y., Yang Y.X., Zhe H., He Z.X., Zhou S.F. (2014). Bardoxolone methyl (CDDO-Me) as a therapeutic agent: an update on its pharmacokinetic and pharmacodynamic properties. Drug Des. Dev. Ther..

[29] Pallesen J.S., Tran K.T., Bach A. (2018). Non-covalent small-molecule kelch-like ECH-associated protein 1-nuclear factor erythroid 2-related factor 2 (Keap1-Nrf2) inhibitors and their potential for targeting central nervous system diseases. J. Med. Chem..

[30] Jiang Z., Lu M., You Q.D. (2016). Discovery and development of kelch-like ECH-associated protein 1: nuclear factor erythroid 2-related factor 2 (KEAP1:NRF2) protein-protein interaction inhibitors: achievements, challenges and future directions. J. Med. Chem..

[31] Magesh S., Chen Y., Hu L. (2012). Small molecule modulators of keap1-nrf2-ARE pathway as potential preventive and therapeutic agents. Med. Res. Rev..

[32] Davies T.G., Wixted W.E., Coyle J.E., Griffiths-Jones C., Hearn K., McMenamin R., Norton D., Rich S.J., Richardson C., Saxty G., Willems H.M.G., Woolford A.J.A., Cottom J.E., Kou J.-P., Yonchuk J.G., Feldser H.G., Sanchez Y., Foley J.P., Bolognese B.J., Logan G., Podolin P.L., Yan H., Callahan J.F., Heightman T.D., Kerns J.K. (2016). Monoacidic inhibitors of the kelch-like ECH-associated protein 1: nuclear factor erythroid 2-related factor 2 (KEAP1:NRF2) protein–protein interaction with high cell potency identified by fragment-based discovery. J. Med. Chem..

[33] Jiang C.S., Zhuang C.L., Zhu K., Zhang J., Muehlmann L.A., Figueiro Longo J.P., Azevedo R.B., Zhang W., Meng N., Zhang H. (2018). Identification of a novel small-molecule Keap1-Nrf2 PPI inhibitor with cytoprotective effects on LPS-induced cardiomyopathy. J. Enzym. Inhib. Med. Chem..

[34] Meng N., Tang H., Zhang H., Jiang C., Su L., Min X., Zhang W., Zhang H., Miao Z., Zhang W., Zhuang C. (2018). Fragment-growing guided design of Keap1-Nrf2 protein-protein interaction inhibitors for targeting myocarditis. Free Radic. Biol. Med..

[35] Jiang Z.-Y., Lu M.-C., Xu L.L., Yang T.-T., Xi M.-Y., Xu X.-L., Guo X.-K., Zhang X.-J., You Q.-D., Sun H.-P. (2014). Discovery of potent keap1–nrf2 protein–protein interaction inhibitor based on molecular binding determinants analysis. J. Med. Chem..

[36] Lu M.-C., Ji J.-A., Jiang Y.-L., Chen Z.-Y., Yuan Z.-W., You Q.-D., Jiang Z.-Y. (2016). An inhibitor of the Keap1-Nrf2 protein-protein interaction protects NCM460 colonic cells and alleviates experimental colitis. Sci. Rep..

[37] Jiang Z.Y., Xu L.L., Lu M.C., Chen Z.Y., Yuan Z.W., Xu X.L., Guo X.K., Zhang X.J., Sun H.P., You Q.D. (2015). Structure-activity and structure-property relationship and exploratory in vivo evaluation of the nanomolar keap1-nrf2 protein-protein interaction inhibitor. J. Med. Chem..

[38] Yoon H.Y., Kang N.I., Lee H.K., Jang K.Y., Park J.W., Park B.H. (2008). Sulforaphane protects kidneys against ischemia-reperfusion injury through induction of the Nrf2-dependent phase 2 enzyme. Biochem. Pharmacol..

[39] Kim J., Cha Y.N., Surh Y.J. (2010). A protective role of nuclear factor-erythroid 2-related factor-2 (Nrf2) in inflammatory disorders. Mutat. Res..

[40] Nath K.A., Vercellotti G.M., Grande J.P., Miyoshi H., Paya C.V., Manivel J.C., Haggard J.J., Croatt A.J., Payne W.D., Alam J. (2001). Heme protein-induced chronic renal inflammation: suppressive effect of induced heme oxygenase-1. Kidney Int..

[41] Lin X., Yang H., Zhou L., Guo Z. (2011). Nrf2-dependent induction of NQO1 in mouse aortic endothelial cells overexpressing catalase. Free Radic. Biol. Med..

[42] Zoete V., Rougee M., Dinkova-Kostova A.T., Talalay P., Bensasson R.V. (2004). Redox ranking of inducers of a cancer-protective enzyme via the energy of their highest occupied molecular orbital. Free Radic. Biol. Med..

[43] Dickinson D.A., Levonen A.L., Moellering D.R., Arnold E.K., Zhang H., Darley-Usmar V.M., Forman H.J. (2004). Human glutamate cysteine ligase gene regulation through the electrophile response element. Free Radic. Biol. Med..

[44] Harvey C.J., Thimmulappa R.K., Singh A., Blake D.J., Ling G., Wakabayashi N., Fujii J., Myers A., Biswal S. (2009). Nrf2-regulated glutathione recycling independent of biosynthesis is critical for cell survival during oxidative stress. Free Radic. Biol. Med..

[45] Rojo de la Vega M., Chapman E., Zhang D.D. (2018). NRF2 and the hallmarks of cancer. Cancer Cell.

[46] Niedernhofer L.J., Daniels J.S., Rouzer C.A., Greene R.E., Marnett L.J. (2003). Malondialdehyde, a product of lipid peroxidation, is mutagenic in human cells. J. Biol. Chem..

[47] Melnikov V.Y., Faubel S., Siegmund B., Lucia M.S., Ljubanovic D., Edelstein C.L. (2002). Neutrophil-independent mechanisms of caspase-1- and IL-18-mediated ischemic acute tubular necrosis in mice. J. Clin. Investig..

[48] Malle E., Buch T., Grone H.J. (2003). Myeloperoxidase in kidney disease. Kidney Int..

[49] Bochkov V.N., Oskolkova O.V., Birukov K.G., Levonen A.L., Binder C.J., Stockl J. (2010). Generation and biological activities of oxidized phospholipids. Antioxidants Redox Signal..

[50] Jha V., Garcia-Garcia G., Iseki K., Li Z., Naicker S., Plattner B., Saran R., Wang A.Y., Yang C.W. (2013). Chronic kidney disease: global dimension and perspectives. Lancet.

[51] Figueira T.R., Barros M.H., Camargo A.A., Castilho R.F., Ferreira J.C., Kowaltowski A.J., Sluse F.E., Souza-Pinto N.C., Vercesi A.E. (2013). Mitochondria as a source of reactive oxygen and nitrogen species: from molecular mechanisms to human health. Antioxidants Redox Signal..

[52] Guan T., Song J., Wang Y., Guo L., Yuan L., Zhao Y., Gao Y., Lin L., Wang Y., Wei J. (2017). Expression and characterization of recombinant bifunctional enzymes with glutathione peroxidase and superoxide dismutase activities. Free Radic. Biol. Med..

[53] Fujita H., Fujishima H., Takahashi K., Sato T., Shimizu T., Morii T., Shimizu T., Shirasawa T., Qi Z., Breyer M.D., Harris R.C., Yamada Y., Takahashi T. (2012). SOD1, but not SOD3, deficiency accelerates diabetic renal injury in C57BL/6-Ins2(Akita) diabetic mice. Metabolism.

[54] Benhar M. (2018). Roles of mammalian glutathione peroxidase and thioredoxin reductase enzymes in the cellular response to nitrosative stress. Free Radic. Biol. Med..

[55] Perez-Estrada J.R., Hernandez-Garcia D., Leyva-Castro F., Ramos-Leon J., Cuevas-Benitez O., Diaz-Munoz M., Castro-Obregon S., Ramirez-Solis R., Garcia C., Covarrubias L. (2019). Reduced lifespan of mice lacking catalase correlates with altered lipid metabolism without oxidative damage or premature aging. Free Radic. Biol. Med..

[56] Glorieux C., Zamocky M., Sandoval J.M., Verrax J., Calderon P.B. (2015). Regulation of catalase expression in healthy and cancerous cells. Free Radic. Biol. Med..

[57] Hwang I., Lee J., Huh J.Y., Park J., Lee H.B., Ho Y.S., Ha H. (2012). Catalase deficiency accelerates diabetic renal injury through peroxisomal dysfunction. Diabetes.

[58] Antunes F., Han D. (2009). Redox regulation of NF-kappaB: from basic to clinical research. Antioxidants Redox Signal..

[59] Sivandzade F., Prasad S., Bhalerao A., Cucullo L. (2019). NRF2 and NF-B interplay in cerebrovascular and neurodegenerative disorders: molecular mechanisms and possible therapeutic approaches. Redox Biol.

[60] Liu G.H., Qu J., Shen X. (2008). NF-kappaB/p65 antagonizes Nrf2-ARE pathway by depriving CBP from Nrf2 and facilitating recruitment of HDAC3 to MafK. Biochim. Biophys. Acta.

[61] Wakabayashi N., Slocum S.L., Skoko J.J., Shin S., Kensler T.W. (2010). When NRF2 talks, who's listening?. Antioxidants Redox Signal..

[62] Bellezza I., Tucci A., Galli F., Grottelli S., Mierla A.L., Pilolli F., Minelli A. (2012). Inhibition of NF-kappaB nuclear translocation via HO-1 activation underlies alpha-tocopheryl succinate toxicity. J. Nutr. Biochem..

[63] Ganesh Yerra V., Negi G., Sharma S.S., Kumar A. (2013). Potential therapeutic effects of the simultaneous targeting of the Nrf2 and NF-kappaB pathways in diabetic neuropathy. Redox Biol..

