# Supplementary material for: CPUY192018, a potent inhibitor of the Keap1-Nrf2 protein-protein interaction, alleviates renal inflammation in mice by restricting oxidative stress and NF-κB activation
Source: Redox Biol. 2019 Jul 2;26:101266. doi: 10.1016/j.redox.2019.101266 (PMC6614503; doi:10.1016/j.redox.2019.101266)
Supplement: Multimedia component 1 [file mmc1.docx]

Supporting Information

CPUY192018, a potent inhibitor of the Keap1-Nrf2 protein-protein interaction, alleviates renal inflammation in mice by restricting oxidative stress and NF-κB activation

Meng-Chen Lu,^a b^ Jing Zhao,^a^ Yu-Ting Liu,^a^ Tian Liu, ^a^ Meng-Min Tao,^a^ Qi-Dong You^a,b,^* and Zheng-Yu Jiang^a,b,^*.

^a^ State Key Laboratory of Natural Medicines and Jiangsu Key Laboratory of Drug Design and Optimization, China Pharmaceutical University, Nanjing 210009, China

^b^ Department of Medicinal Chemistry, School of Pharmacy, China Pharmaceutical University, Nanjing 210009, China.

**Table of contents:**

S1. Synthetic route of **CPUY192018**.

S2. The cytotoxicity of **CPUY192018** against HK-2 cells

**S1. Synthetic route of CPUY192018.**

Scheme S1. Synthetic route of **CPUY192018**.

Amination of commercially available 1-nitronaphthalene afforded 4-Nitronaphthalen-1-amine. The nitro group of **1** was reduced by hydrogen and Pd/C, and subsequent condensation with 4-acetamido benzenesulfonyl chloride gave compound **2**. Compound **3** were obtained by nucleophilic substitution of NH by methyl bromoacetate in the presence of K_2_CO_3_ in DMF. Hydrolysis of the ester groups of **3** yielded the target compound **CPUY192018.**


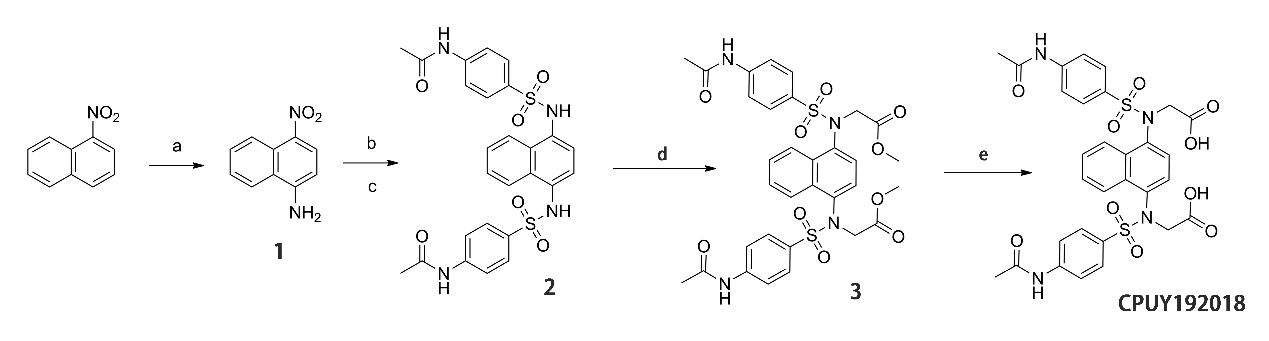


Reagents and conditions: (a) NH_2_OH·HCl, 95% ethanol, MeOH, 60 °C, 2h; (b) Pd/C, H_2_, rt, 4h; (c) 4-acetamido benzenesulfonyl chloride, toluene, pyridine, 100 °C, 2h, 71%; (d) DMF, K_2_CO_3_, methyl bromoacetate, rt, 3h, 54%; (e) LiOH, CH_3_OH/H_2_O, rt, 6h, 68%.

**S2. The cytotoxicity of CPUY192018 against HK-2 cells**

We examined the cytotoxicity of **CPUY192018** against the HK-2 cells using the MTT assay. Briefly, HK-2 cells in logarithmic phase were seeded at the density of 70 ~ 80% confluence per well in 96-well plates at 37°C with 5% CO_2_ for overnight incubation and treated with various concentrations (1.56 – 100 μM) of **CPUY192018** for 48 h. After treatment, 20 μL of 5 mg/mL MTT was added and the cells were incubated for 4 h at 37°C. The supernatant was discarded and 150 μL of DMSO was added to each well. The mixture was shaken on a mini shaker at r.t. for 5 min and the spectrophotometric absorbance was measured by Multiskan Spectrum Microplate Reader (Thermo, USA). Triplicate experiments were performed in a parallel manner for each concentration point. The data in **Figure S1** showed that the survival rate remained higher than approximately 80% under 100 μM, indicating no apparent cytotoxicity for the treatment of **CPUY192018**.





**Figure S1.** The cytotoxicity of **CPUY192018** against the HK-2 cells using the MTT assay.
